# Arterial pulse wave modeling and analysis for vascular-age studies: a review from VascAgeNet

**DOI:** 10.1152/ajpheart.00705.2022

**Published:** 2023-03-31

**Authors:** Jordi Alastruey, Peter H. Charlton, Vasiliki Bikia, Birute Paliakaite, Bernhard Hametner, Rosa Maria Bruno, Marijn P. Mulder, Samuel Vennin, Senol Piskin, Ashraf W. Khir, Andrea Guala, Christopher C. Mayer, Jonathan Mynard, Alun D. Hughes, Patrick Segers, Berend E. Westerhof

**Affiliations:** ^1^Department of Biomedical Engineering, School of Biomedical Engineering and Imaging Sciences, King’s College London, London, United Kingdom; ^2^Department of Public Health and Primary Care, University of Cambridge, Cambridge, United Kingdom; ^3^Division of Vascular Surgery, School of Medicine, Stanford University, Stanford, California, United States; ^4^Laboratory of Hemodynamics and Cardiovascular Technology, Institute of Bioengineering, Swiss Federal Institute of Technology, Lausanne, Switzerland; ^5^Biomedical Engineering Institute, Kaunas University of Technology, Kaunas, Lithuania; ^6^AIT Austrian Institute of Technology, Center for Health and Bioresources, Medical Signal Analysis, Vienna, Austria; ^7^INSERM, U970, Paris Cardiovascular Research Center, Université de Paris, Hopital Europeen Georges Pompidou–APHP, Paris, France; ^8^Cardiovascular and Respiratory Physiology, TechMed Centre, University of Twente, Enschede, The Netherlands; ^9^Department of Mechanical Engineering, Faculty of Engineering and Natural Sciences, Istinye University, Istanbul, Turkey; ^10^Department of Engineering, Durham University, Durham, United Kingdom; ^11^Vall d’Hebron Institut de Recerca, Barcelona, Spain; ^12^CIBER-CV, Instituto de Salud Carlos III, Madrid, Spain; ^13^Heart Research, Murdoch Children’s Research Institute, Parkville, Victoria, Australia; ^14^Department of Paediatrics, University of Melbourne, Parkville, Victoria, Australia; ^15^Department of Biomedical Engineering, University of Melbourne, Parkville, Victoria, Australia; ^16^MRC Unit for Lifelong Health and Ageing at UCL, Department of Population Science and Experimental Medicine, Institute of Cardiovascular Science, University College London, London, United Kingdom; ^17^Institute for Biomedical Engineering and Technology, Ghent University, Ghent, Belgium; ^18^Department of Pulmonary Medicine, Amsterdam University Medical Centres, Vrije Universiteit Amsterdam, Amsterdam, The Netherlands; ^19^Department of Neonatology, Radboud University Medical Center, Radboud Institute for Health Sciences, Amalia Children’s Hospital, Nijmegen, The Netherlands

**Keywords:** aging, arteriosclerosis, atherosclerosis, hemodynamics, pulse wave

## Abstract

Arterial pulse waves (PWs) such as blood pressure and photoplethysmogram (PPG) signals contain a wealth of information on the cardiovascular (CV) system that can be exploited to assess vascular age and identify individuals at elevated CV risk. We review the possibilities, limitations, complementarity, and differences of reduced-order, biophysical models of arterial PW propagation, as well as theoretical and empirical methods for analyzing PW signals and extracting clinically relevant information for vascular age assessment. We provide detailed mathematical derivations of these models and theoretical methods, showing how they are related to each other. Finally, we outline directions for future research to realize the potential of modeling and analysis of PW signals for accurate assessment of vascular age in both the clinic and in daily life.

##  1. INTRODUCTION

Pulse wave (PW) signals are produced by the pumping heart and its interaction with the blood and the distensible arterial walls. Cardiac ejection increases blood pressure and distends the wall of the aorta, generating a compression/distension wave that propagates along the aorta and other conduit arteries. Toward the end of systole, a decline in cardiac ejection rate creates a decompression/relaxation wave that manifests as a decline in pressure and a reduction in aortic diameter. Together, these and other (transmitted and reflected) waves create a waveform that is called the pulse wave. The pulse wave leads to a rhythmical expansion and relaxation of all arteries that follows the heartbeat; e.g., producing the pulse that can be felt in the radial artery of the wrist, despite the wrist being about a meter away from the heart. The pulsatile movement of the arterial wall is accompanied by changes, over time and space, in blood pressure, blood flow velocity, and blood volume flow rate throughout the arterial system, called, respectively, pressure, flow velocity, and flow rate PWs.

PW signals can be measured in vivo using a variety of (invasive and noninvasive) devices and are influenced by the heart and the vasculature, making them a rich source of information on cardiovascular (CV) health. In particular, the morphology of PW signals is affected by changes in the mechanical and structural properties of the vascular wall produced by vascular aging or disease, and their impact on cardiac mechanics and structure. Vascular aging is a complex biological process that involves the deterioration in structure and function of blood vessels over time and may occur at a different rate than chronological aging (1). It is a critical component of overall aging that entails an increase in arterial wall stiffness (arteriosclerosis) and the accumulation of atheroma that results in progressive narrowing of the arterial lumen (atherosclerosis) (2, 3). Initially, vascular deterioration is usually an asymptomatic process that eventually can cause damage to the heart, brain, kidneys, and other organs. Measures of vascular age encompass the cumulative effect of all CV risk factors on the arterial wall throughout life (3). Therefore, assessment of vascular age by PW analysis may help identify individuals with early vascular aging (4), and, hence, at elevated CV risk, at an early stage of disease progression.

Arterial PW modeling and analysis aims to unravel the functioning of the CV system through the measurement, mathematical analysis, and computational and experimental simulation of pulsatile hemodynamics (i.e., the dynamics of pulsatile blood flow). In addition to the widely used values of systolic, diastolic, and mean arterial pressure, other clinically relevant information for vascular age assessment can be derived from the morphology of PW signals. As shown in this review, several PW analysis techniques can provide multiple hemodynamic measures and indices that vary with aging and disease, suggesting that they may constitute relevant indicators of age-related CV risk. Models for simulating PWs can be used to investigate the accuracy of these techniques, provide mechanistical insights, and understand the physiological basis underlying measured hemodynamics phenomena. However, PW models should be developed further to better capture the diversity of PW measurements observed in vivo and combined with artificial intelligence (AI) for an improved assessment of vascular aging in daily life.

This article reviews the possibilities, limitations, complementarity, and differences of reduced-order, arterial PW models (*Section 2*) and analysis methods (*Section 3*) for CV assessment, with a focus on vascular age assessment. It aims to provide a comprehensive overview of models and analytical techniques to help someone new in the field get started, including engineers, mathematicians, and physicians, as well as to be a convenient compendium for established researchers. Directions for future research in the field are also provided (*Section 4*). This article is free of mathematical derivations and equations to make it accessible to readers with a limited mathematical background. It is accompanied by a Technical Supplement (https://doi.org/10.6084/m9.figshare.21758012.v3) containing technical details and mathematical derivations of all the biophysical models and hemodynamics-based analysis techniques covered in the article. All derivations start from the well-known Navier–Stokes equations, to show how different types of models and analysis techniques are related to each other.

## 2. PULSE WAVE MODELS

**Figure 1.**
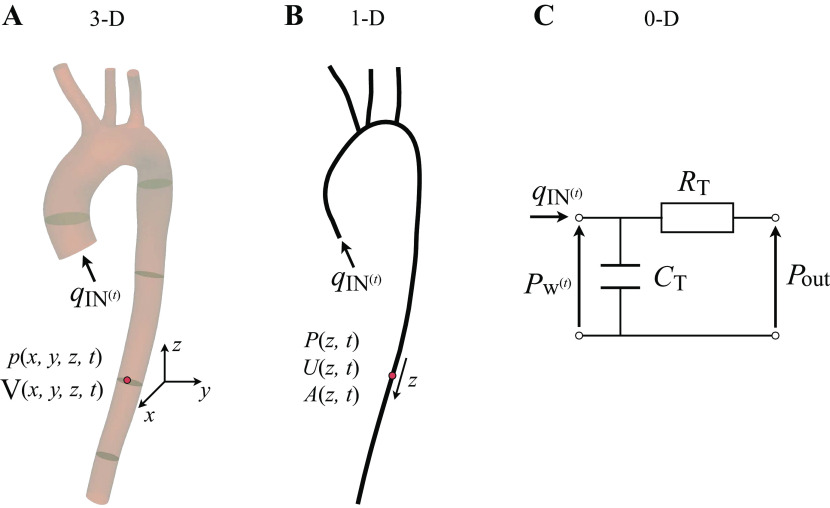
The main arterial blood flow modeling approaches illustrated for the upper aorta. *A*: three-dimensional (3-D) models simulate blood pressure (*p*), blood flow velocity (*V*), and wall displacement (not shown) as a function of time (*t*) and three spatial dimensions (e.g., *x*, *y*, and *z* in Cartesian coordinates). *B*: one-dimensional (1-D) models describe blood pressure (*P*), blood flow velocity (*U*), and luminal area (*A*) with time and axial direction of the vessel (*z*). *C*: zero-dimensional (0-D) models can calculate a space-independent blood pressure (*p*_w_) for the whole 3-D or 1-D arterial tree as a function of the aortic inflow (*q*_IN_), total compliance (*C*_T_) and resistance (*R*_T_), and outflow pressure (*P*_out_) at each terminal segment of 3-D and 1-D models (see *Eq. 80* in Technical Supplement).

Arterial hemodynamics obeys physical laws and principles (i.e., conservation of mass, momentum, and energy) that can be used to mathematically describe (i.e., model) arterial PW signals. There are three main physics-based modeling approaches—zero-dimensional (0-D), one-dimensional (1-D), and three-dimensional (3-D) models—that are illustrated in Fig. 1 and compared in Table 1 in terms of their spatial accuracy, computation time, and advantages and limitations to study vascular aging. Large-scale network simulations of PW signals often require simplification of the 3-D formulation to reduce the computation time while maintaining reasonable accuracy. This can be achieved using the reduced-order 1-D (*Section 2.1*) and 0-D (*Section 2.2*) models. There are only a few commercial software programs for PW modeling. One such program is called the “Aplysia CardioVascular Lab” (Aplysia Medical AB, Stockholm, Sweden), which features a basic 0-D model arterial network that enables users to simulate both central and peripheral PWs (5).

**Table 1.** Comparison of the main arterial blood flow modeling approaches

Model	Spatial Accuracy	Computation Time	Advantages	Limitations
0-D	Space-independent blood pressure, blood flow, and luminal volume	Seconds	Computationally inexpensive descriptions of *1*) global blood flow features altered by VA (e.g., systemic arterial compliance) in the whole cardiovascular system and *2*) boundary conditions for 1-D and 3-D models	Inability to describe *1*) high-frequency PW features, *2*) PW propagation phenomena, and *3*) spatial variations in vessel geometric and material properties altered by VA
1-D	Crossed-sectionally averaged blood pressure and blood flow velocity, and luminal area along the vessel’s axis	Seconds to minutes	Good trade-off between accuracy and computation time to *1*) describe PW signals in large-scale networks, accounting for wave reflection and transmission effects and spatial variations in vessel geometric and material properties altered by VA and *2*) improve boundary conditions for 3-D models	Inability to describe *1*) complex blood flow phenomena with non-negligible radial and circumferential flows due to VA-related structural changes (e.g., aneurysms and stenoses), *2*) the mechanical stresses these flows produce on the arterial wall, and *3*) blood flow in the microcirculation
3-D	Blood pressure, blood flow velocity, and arterial wall displacements in three dimensions	Hours to days	Description of complex local blood flow phenomena and the mechanical stresses they produce on the arterial wall, with a high level of geometrical, structural, and biophysical detail	Computationally expensive and reliance on detailed input data that can be challenging or even impossible to acquire (e.g., regional stiffness)

Zero-dimensional (0-D), one-dimensional (1-D), and three-dimensional (3-D) models can all describe time-varying pulse wave (PW) signals. However, they have different degrees of spatial accuracy and computation time, which determine their advantages and limitations to study vascular aging (VA).

### 2.1. 1-D Models

One-dimensional models of the arterial tree are considered a good compromise between accuracy and computational cost for simulating arterial PW signals. The inviscid 1-D governing equations of conservation of mass and momentum were derived by Leonhard Euler in 1755 (6). Other historical figures that made important contributions to the field of 1-D blood flow modeling include W. Weber, T. Young, J. L. Poiseuille, B. Riemann, and J. R. Womersley. For a historical overview see Parker (7), the PhD theses of Westerhof (8) and Hughes (9), and the introductions of the articles by Hughes and Lubliner (10), and van de Vosse and Stergiopulos (11). The 1-D model formulation is described in *Section 2.1.1*, followed by an overview of how to calibrate model parameters (*Section 2.1.2*), verify the accuracy of simulated PW signals (*Section 2.1.3*), and use 1-D models to study clinically relevant problems for vascular aging (*Section 2.1.4*).

#### 2.1.1. Formulation.

In 1-D modeling, the arterial network is described as a set of arterial segments interconnected at nodes (Fig. 1*B*). Within each segment, blood pressure, blood flow velocity, and luminal cross-sectional area vary with time and distance along the axis of the vessel, governed by a system of partial differential equations. The governing equations ensure that the *1*) physical principles of conservation of mass and linear momentum for blood flow are satisfied in each arterial segment and *2*) interaction between blood flow and vessel wall deformations is accounted for.

Technical Supplemental Section 2.1.1 provides a detailed derivation of the 1-D governing equations starting from the 3-D Navier–Stokes equations in cylindrical coordinates, based on the work of Barnard et al. (12), and involving the solid mechanics theory of thin-walled pressure vessels. Radial and azimuthal variations in blood pressure and flow velocity, which are considered in 3-D modeling, are neglected in 1-D modeling to reduce complexity and computational cost. This is achieved by *1*) assuming cylindrical symmetry to eliminate azimuthal variations *Eqs. 2*, *11*, and *12*^1^, *2*) assuming that axial blood flow velocities are much larger than radial velocities to eliminate secondary terms in the equations (i.e., considering ε ≪ 1 in *Eqs. 15* and *16*), and *3*) integrating over the luminal cross section to eliminate radial variations (*Eqs.* 3 and 22). The second point follows from the long-wave approximation: arterial pulse wavelengths are much longer (of the order of meters) than vessel wall displacements in the radial direction (≪1 cm) (*Eq. 17*). Additional assumptions include fixed-length and longitudinally tethered vessels, incompressible and Newtonian fluid^2^, and fully developed laminar^3^ flow.

Arterial wall models in 1-D modeling describe the relation between pressure and cross-sectional area. They are referred to as tube laws. These range from purely elastic laws in which the vessel wall elasticity (which decreases with vascular aging) is described by Young’s modulus, to more complex laws that account for nonlinear elastic behavior (16), stress relaxation (17–19), wall viscosity (20–22), and wall inertia (16, 23). Technical Supplemental 2.1.1.4 shows how to derive an elastic tube law and extend this law to include wall viscosity.

At the arterial junctions of the arterial network, a junction problem needs to be solved, usually by enforcing the conservation of mass and energy (24), although more complex approaches that account for pressure losses at junctions are also available (25). In addition, appropriate boundary conditions need to be prescribed at the inflow and outflow arterial segments. At the inflow (usually the aortic root), the flow waveform is often enforced (see, e.g., Refs. 26–28). Alternatively, 0-D models of cardiac contraction can be coupled to the aortic root if the 1-D model network starts there (see *Section 2.2.3*). Any 1-D model network has to be truncated after a few generations of bifurcations. Indeed, care should be taken when simulating blood vessels with diameters smaller than 1 mm since the assumptions of blood being a continuum and Newtonian fluid start failing as the relative size of red blood cells to vessel diameter increases. Terminal 1-D model branches are often coupled to 0-D models relating the flow to pressure at the branch’s endpoint and accounting for physical properties of the downstream vasculature (see, e.g., Refs. 29–31). More sophisticated terminal models include single tapering vessels (32), structured-tree networks (27, 33–35), and open-loop or closed-loop 0-D compartmental models. Structured-tree models can be used to investigate the effects of small-vessel vascular disease, such as stiffening and rarefaction (36), and to predict flow and pressure profiles in the microvasculature. Compartmental 0-D models can describe the peripheral circulation, venous return, pulmonary circulation, and heart chambers; therefore bridging the inflow and outflow boundaries of the 1-D model arterial network and simulating the entire circulation as a closed-loop computational domain (31, 37–41).

Table 2 compares the main characteristics of existing 1-D models for simulating PW signals. These range from single-vessel models (e.g., of the aorta) to closed-loop models of the entire circulation, including the four heart chambers. The aorta and other larger arteries of the head, thorax, abdomen, and upper and lower limbs are often included, and a few models also account for the larger arteries of the pulmonary, coronary, and/or cerebral circulations. Earlier models focused on simulating a few arteries of systemic circulation, with special attention paid to the cerebral arteries. Existing 1-D formulations may differ on the tube law used and the way velocity profiles, convective accelerations, and distal vasculatures are simulated.

**Table 2.** Main characteristics of existing 1-D models

References		Closed-Loop	Heart Model	Systemic Circulation	Pulmonary Circulation	Coronary Circulation	Cerebral Circulation	Tube Law	Velocity Profile	Convective Acceleration	Distal Vasculature Models
Streeter et al.	(42)	−	−	− (aorta)	−	−	−	NLE	− P	−	R
Schaaf and Abbrecht	(43)	−	−	+	−	−	−	LE	− P	−	R
Wemple and Mockros	(44)	−	−	+	−	−	−	NLE	+ W	+	3Wk
Raines et al.	(45)	−	−	− (leg)	−	−	−	NLE	− P	−	3Wk
Avolio	(46)	−	−	+	−	−	+	VE	+ W	−	R
Stettler et al.	(47, 48)	−	−	+	−	+	−	NLE	− P	−	R
Kufahl and Clark	(49)	−	−	− (cerebral)	−	−	+	NLE	− p	+	3Wk
Hillen et al.	(50)	−	−	− (cerebral)	−	−	+	LE	− P	−	R
Papapanayotou et al.	(51)	−	−	− (cerebral)	−	−	+	LE	− P	−	R
Fitchett	(52)	−	+1 C	+	−	−	+	VE	+ W	−	R
Stergiopulos et al.	(29)	−	−	+	−	−	−	NLE	− P	+	3Wk
Cassot and Zagzoule	(53)	−	−	− (cerebral)	−	−	+	LE	− P	−	R
Olufsen	(33)	−	−	+	−	−	−	LE	+ BL	−	ST
Wan et al.	(54)	−	−	+	−	−	−	LE	− P	+	R
Sherwin et al.	(55)	−	−	+	−	−	−	LE	− F	−	R
Wang and Parker	(56)	−	−	+	−	−	−	LE	− F	−	R
Formaggia et al.	(30)	−	+1 C	+	−	−	−	LE	− P	−	3Wk
Azer and Peskin	(34)	−	−	+	−	−	−	LE	+ W	+	ST
Bessems et al.	(57)	−	−	− (aorta and coronary)	−	−	−	LE	+ BL	+	3Wk
Huo and Kassab	(35)	−	−	− (coronary)	−	+	−	LE	− P	+	ST
Liang et al.	(38)	+	+4 C	+	+	−	−	LE	− p	−	Wk+
Reymond et al.	(21)	−	+1 C	+	−	+	+	VE	+ W	+	3Wk
Blanco et al.	(26)	−	−	+	−	+	+	VE^1^	− P	−	3Wk
Müller and Toro	(31)	+	+4 C	+	−	−	+	LE	− P	−	Wk+
Qureshi et al.	(27)	−	−	−	+	−	−	LE	+ BL	−	ST
Mynard and Smolich	(39)	+	+4 C	+	+	+	+	VE^3^	− p	−	Wk+^2^
Acosta et al.	(58)	+	+4 C	+	+	−	−	LE	− p	−	Wk+^2^
Carson et al.	(59)	+	+4 C	+	+	+	−	VE	+ BL	−	3Wk+
Charlton et al.	(28)	−	−	+	−	−	+	VE	− p	−	3Wk
Gallo et al.	(41)	+	+4 C	+	−	−	+	VE	+ BL	−	3Wk+
Westerhof et al.	(60)	−	−	+	−	−	+	VE	− P	−	3Wk

Closed-loop: a closed-loop model of the circulation is (+) or not (−) included. Heart model: a heart model is (+) or not (−) coupled to the one-dimensional (1-D) model vessels, with the number of heart chambers (C) indicated. Systemic circulation: the larger systemic arteries are simulated using 1-D modeling (+), as opposed to none or a few arteries as indicated (−). Pulmonary circulation: the larger pulmonary arteries are simulated using 1-D modeling (+), as opposed to none or a few arteries (−). Coronary circulation: the larger coronary arteries are simulated using 1-D modeling (+), as opposed to none or a few arteries (−). Cerebral circulation: the larger cerebral arteries, including the circle of Willis, are simulated using 1-D modeling (+), as opposed to none or a few arteries (−). Tube law: Arterial wall modeled as a linear (LE) or nonlinear (NLE) purely elastic material, or as a viscoelastic (VE) material. Velocity profile: profile calculated (+) using Womersley flow (W) or a boundary layer method (BL), or prescribed (−) using Poiseuille flow (P), a higher-order polynomial (p), a boundary layer method (BL), or a flat profile (F). Convective acceleration: Full term simulated (+) or either simplified by assuming a flat velocity profile or completely neglected (−). Distal vasculature models: single resistance (R), three-element Windkessel (3Wk), Windkessel with more than three elements (Wk+), structured-tree (ST).

1 Elastin, collagen, smooth muscle contributions accounted for; ^2^Nonlinear 0-D models, with specific models for the hepatic and coronary beds; ^3^Nonlinear elastic term using a power law and Voigt-type viscous term.

Recently, the photoplethysmogram (PPG) signal [an optical measure of the arterial PW that can be measured in daily life (61)] has been simulated using 1-D modeling; either calculated *1*) as being proportional to arterial blood volume in a vascular bed (28) or luminal area (62) or *2*) from the simulated pressure wave using a transfer function (63).

#### 2.1.2. Calibration.

Arterial PW models are composed of sets of equations, each of which comprises a number of parameters that need to be specified. This is often referred to as calibration. A particular set of parameters allows for the simulation of physiologically realistic PWs representative of a particular subject or pathology. Baseline 1-D models have been calibrated to simulate PWs representative of young, healthy (21, 39) and male subjects (28). These baseline models have then been personalized to simulate PWs for specific subjects (64–66) and adapted to model changes that occur with aging (28, 60, 67–70) (summarized in Table 3), hypertension (24) and its treatment (80), aneurysms (81, 82), stenoses (83, 84), and variability within a cohort (28, 85). The following model parameters are typically calibrated, whereas others are held constant as they have less effect on PWs (28): arterial geometry (length and diameter of each arterial segment), arterial stiffness, flow from the left ventricle into the aorta, and microvascular properties (resistance, compliance, and outflow pressure). The methods used to calibrate models are described in Technical Supplemental Section 2.1.2.

**Table 3.** Changes in mechanical and structural properties of the cardiovascular system with chronological age

Parameter	Age Variation	References
Heart rate	Nonlinear change in M/F	(71)
Stroke volume	Dec. in M/F	(72)
Cardiac output	Dec. by 24% (M) and 7% (F) between 20 and 69 yr old	(73)
Left ventricular ejection time	No change	(74)
End-systolic elastance	Inc. by 51% between 20 and 80 yr old to normalize left ventricular stress (F > M)	(70, 75)
End-diastolic elastance	Inc. by 51% between 20 and 80 yr old to normalize left ventricular stress (F > M)	(70, 75)
Arterial ventricular coupling	Dec. slightly in F; no change in M	(75)
Arterial length	Inc. in proximal aorta length; no change in the lengths of other arterial segments	(76)
Arterial diameter	Inc. in aortic and carotid diameters; no change in the diameters of other arterial segments	(76, 77)
Arterial stiffness	Nonlinear inc.	(78)
Arterial tree compliance	Dec.	(79)
Peripheral vascular resistance	Inc. or no change	(28)

Variations are given for adult males (M) and females (F) and in childhood if available. dec., decrease; inc., increase.

**Figure 2.**
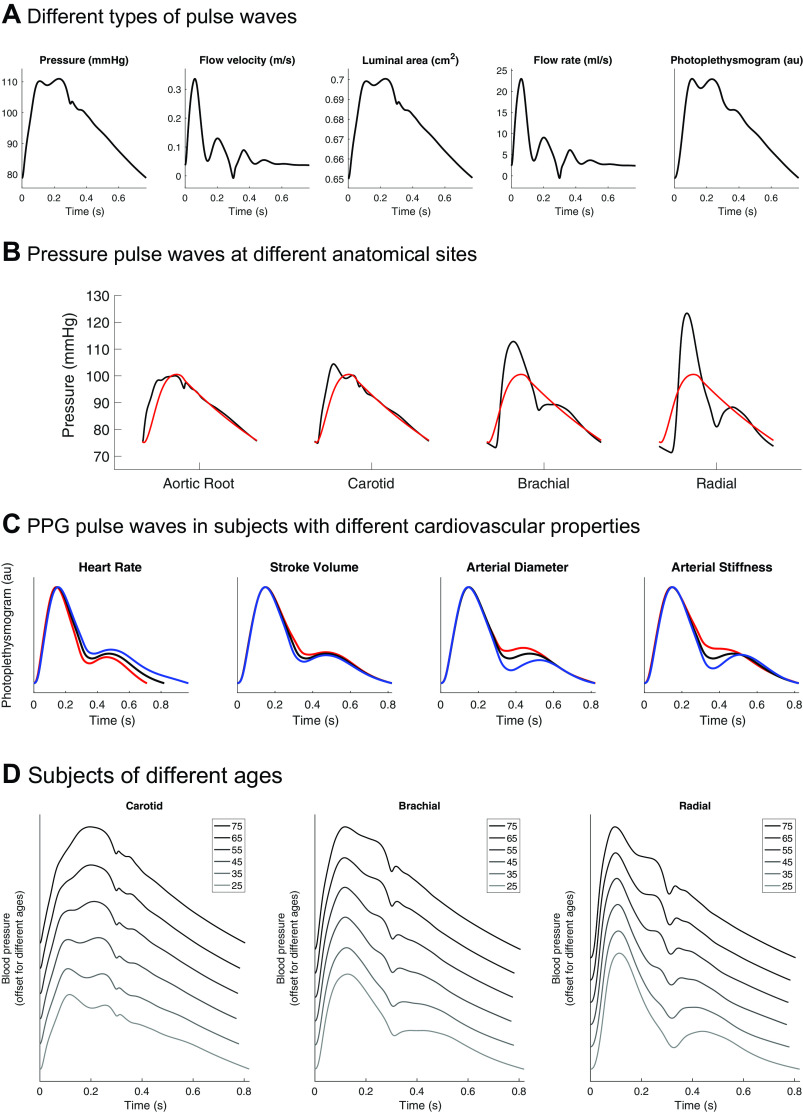
One-dimensional (1-D) blood flow modeling used to simulate arterial pulse waves (PWs). *A*: simulation of different types of PWs at the carotid artery. *B*: simulation of pressure PWs at different anatomical sites together with the analytical zero-dimensional (0-D) pressure (red) given by *Eq. 80* (Technical Supplement). *C*: simulation of photoplethysmogram (PPG) PWs at the wrist for subjects with different cardiovascular properties (black, baseline; red, increase; blue, decrease). *D*: simulation of pressure PWs for subjects of different ages. Source: data were obtained from the Pulse Wave Database (28, 86).

**Figure 3.**
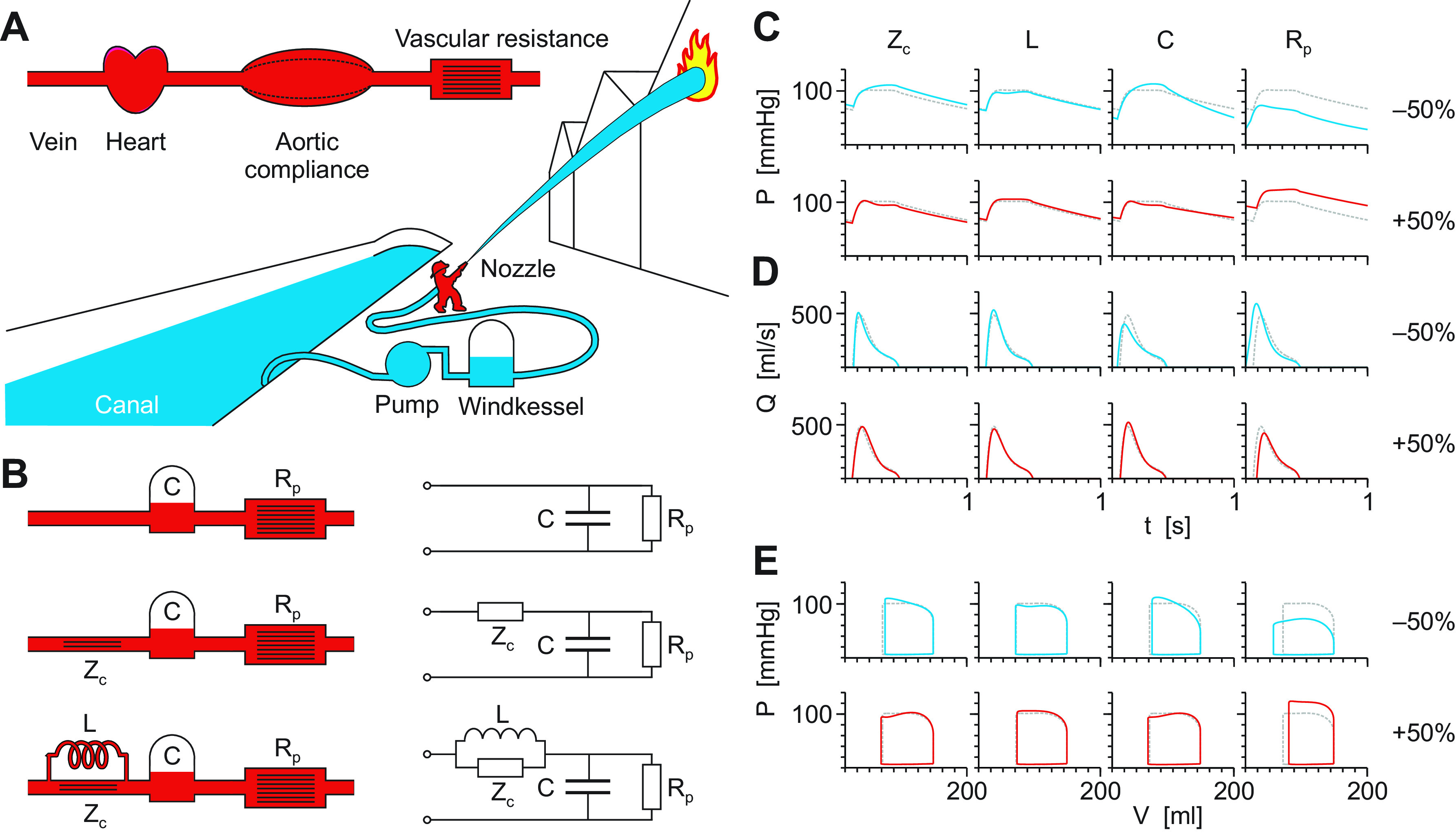
Zero-dimensional Windkessel models of the systemic circulation: fire engine analogy (*A*), hydraulic (*left*) and electrical circuit (*right*) analogies (*B*); blood pressure (*P*; *C*), and blood flow (*Q*; *D*) with time, and pressure-volume (*P*-*V*; *E*) loops simulated using the four-element model (131, 144) with independent increases (red) or decreases (blue) by ±50% in characteristic impedance (*Z*_c_), inductance (*L*), compliance (*C*), and peripheral resistance (*R*_p_), from the baseline model (gray). Changes in *Z*_c_ alter pressure wave shape, with decreased *Z*_c_ causing late systolic peaking. Variations in *L* have limited impact, with a slight flattening of systolic *P* observed when *L* is low. Changes in *C* affect pulse pressure and systolic peak timing, whereas decreased *R*_p_ lowers overall *P* and causes earlier ejection. Higher pressures result in lower flows, and changes in *P* vs. *Q* appear as “mirrored” alterations. The alterations in *P*, such as changes in pulse pressure and early or late systolic peaks, can also be seen in *P*-*V* loops.

**Figure 4.**
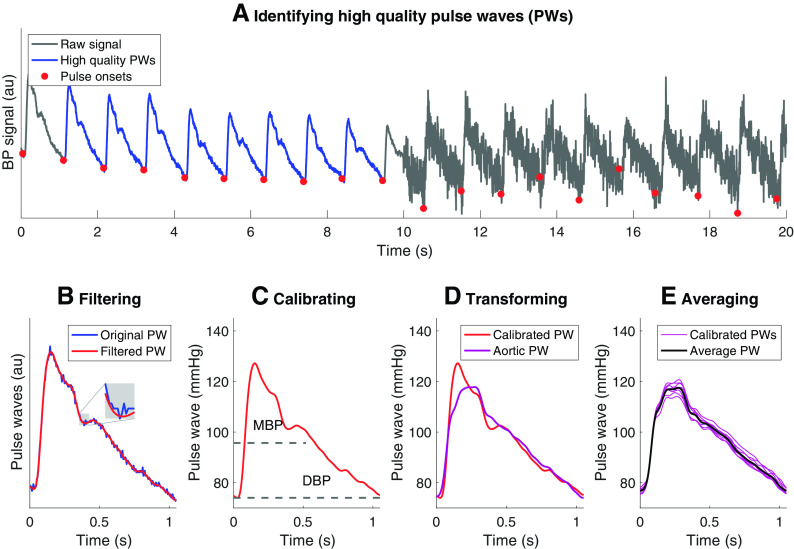
Preprocessing an arterial pulse wave (PW) signal. *A*: brachial applanation tonometry blood pressure (BP) signal was processed to identify high-quality PW data for analysis (blue, from 1 to 9 s). PW onsets are detected (indicated by red dots). *B*: individual PWs are filtered to eliminate high-frequency content. *C*: PWs are calibrated using independent mean and diastolic blood pressure measurements (MBP and DBP, respectively). *D*: brachial (peripheral) PWs are transformed to aortic (central) PWs. *E*: PWs are ensemble averaged to produce a final PW for analysis. Sources: (data) Brachial data from the Asklepios data set, with artificial noise added (334); (processing) PulseAnalyse (28, 192).

Recently, 1-D models have been used to simulate PWs for a set of virtual subjects representative of a population sample. For instance, in Refs. 28 and 85, PWs were simulated to mimic those that would be measured from samples of healthy adults of different ages. This was performed in three steps: *1*) suitable values for parameters were identified from the literature, including mean values for each age group, and ranges of variability within each age group; *2*) these values were converted into model parameters where necessary (such as converting reported PW velocities into Young’s moduli); and *3*) model parameters were adjusted where necessary to provide more realistic PWs. As illustrated in Fig. 2, this approach allows different types of PWs to be simulated, at a range of anatomical sites, for subjects with different CV properties, and different ages. The morphology of these waves matches in vivo data showing, for instance, *1*) a similarity among pressure, luminal area and PPG signals, and between flow velocity and flow rate signals (Fig. 2*A*); *2*) pulse pressure (PP, the amplitude of the pressure PW) amplification from central to peripheral anatomical sites (Fig. 2*B*); *3*) an earlier arrival time of the diastolic peak in the PPG signal with increased arterial stiffness (Fig. 2*C*), and *4*) increases in PP with aging (Fig. 2*D*).

#### 2.1.3. Verification.

Arterial PW models can be verified by comparing the model outputs with reference in silico, in vitro, and in vivo data (see Table 4). Simulated PWs have been compared with reference PWs by qualitatively assessing their shapes and PW-derived indices such as mean blood pressures and flow rates (21, 28, 39, 64, 65, 84, 95, 97–100). Error statistics have been used to quantify the performance of models: statistics such as the (relative) root mean square error (RMSE; see, for example, Refs. 64 and 90) and relative (or percentage) error (90) quantify the overall performance of model simulations (see Technical Supplemental Section 2.1.3). Quantitative comparisons have shown relative RMSEs between 1-D model and reference PWs of as little as 1.2% for pressure, 2.1% for the flow, and 2.6% for the luminal cross-sectional area (see Table 4). In these studies, reference PWs included those measured in well-defined CV simulation rigs made of flexible tubes (18, 92–95) and those computed using 3-D fluid-structure interaction models with identical boundary conditions and compatible geometrical and material properties (32, 88, 89, 92). A few comparisons in diseased vasculature with stenosis and aneurysms have also been carried out (84, 92, 98). All these studies show that 1-D modeling can simulate PWs in large arteries, in steady state, supine conditions, and over one cardiac cycle, with a reasonable computational cost and with accuracies comparable with those obtained by 3-D models.

**Table 4.** Review of studies assessing the accuracy of 1-D model pulse waveforms

References		Test Data	Simulated Arteries	ε_P_	ε_Q_	ε_U_	ε_A_
Mynard et al.	(32)	3-D data	Carotid bifurcation	-	-	*	-
Reymond et al.	(87)	3-D data	Upper Ao and supra Ao arteries	*	*	-	-
Grinberg et al.	(88)	3-D data	50 larger intracraneal arteries	*	*	-	-
Xiao et al.	(89)	3-D data	CCA, thoracic Ao, aortic bif.	1.4	2.1	-	2.6
Xiao et al.	(89)	3-D data	20 larger sys. arteries	2.1	4.9	-	-
Boileau et al.	(90)	3-D data	CCA, thoracic Ao, aortic bif.	1.2	2.6	-	4.3
Alastruey et al.	(91)	3-D data	Upper Ao and supra Ao arteries	2.0	5.0	-	3.0
Jin and Alastruey	(92)	3-D data	Abdominal Ao, carotid and iliac§	5.4	7.3	-	-
Bessems et al.	(18)	in vitro	Ao†	*	*	-	-
Alastruey et al.	(93)	in vitro	37 larger sys. arteries	2.5	10.8	-	-
Saito et al.	(94)	in vitro	9 larger sys. arteries	10.0	*	-	-
Huberts et al.	(95)	in vitro	Upper-limb arteries	*	*	-	-
Boileau et al.	(90)	in vitro	37 larger sys. arteries	4.0	25.6	-	-
Jin and Alastruey	(92)	in vitro	Ao	5.0	-	-	-
Avolio	(46)	human	128 larger sys. arteries	-	-	*	-
Stettler et al.	(47, 48)	human	Ao and lower limb arteries	*	*	-	-
Olufsen et al.	(96)	human	29 larger sys. arteries	-	*	-	-
Reymond et al.	(21)	human	103 larger sys. arteries	*	*	-	-
Reymond et al.	(64)	human	94 larger sys. arteries	6.0‡	11.0	-	-
Willemet et al.	(97)	human	Lower-limb arteries	9.6	-	16.0	-
Guala et al.	(65)	human	Larger sys. arteries	13.0	-	-	-
Mynard and Smolich	(32)	human	Larger sys. and pul. arteries	*	*	*	-
Alastruey et al.	(91)	human	Upper Ao and supra Ao arteries	10.0	7.0	-	8.0
Strocchi et al.	(84)	human	55 larger sys. arteries	*	*	-	-
Charlton et al.	(28)	human	116 larger sys. arteries	*	*	-	-
Steele et al.	(98)	animal	Aortic bypass	-	4.2	-	-
Mynard et al.	(99)	animal	Left conduit coronary arteries	-	16.7	-	-

The third column shows the type of reference data used in each study. Upper bounds for relative errors (in percentage) for pressure (ε_P_), flow rate (ε_Q_), flow velocity (ε_U_) and cross-sectional area (ε_A_) wave morphology, calculated as described in the corresponding article, are shown when available. (Adapted from Ref. 92). Ao, aorta; bif., bifurcation; CCA, common carotid artery; pul., pulmonary; sys., systemic. -, no comparison made.

*Qualitative comparison; †according to the dimensions shown in Fig. 4 of (18); ‡except at the abdominal aorta, where root mean square error is 21%; §for stenosis and aneurysm sizes of up to 85% and 400%, respectively.

The ability of 1-D models to accurately simulate PWs and precisely mimic changes in PWs under changing CV conditions are both of interest. Statistical relationships between continuous measures derived from PW models are commonly examined using correlation, under the assumption of linear (Pearson’s) or monotonic (Spearman’s) relationships between variables to produce quantitative estimates of dependency. Information theory-based metrics, such as mutual information that quantifies all the dependencies between two variables (not just linear or rank dependencies) (101), have also been used in studies of PWs (102, 103) but seem not to have been used as a measure of fidelity. In Refs. 66 and 104, the accuracy and precision of simulated blood pressures were assessed using the bias (i.e., mean error) and limits of agreement (i.e., range around the bias within which 95% of errors are expected to fall), respectively: this separation allows assessment of the suitability of a model for simulating PWs for an individual at a given time and simulating changes in PWs either between individuals or within an individual over time.

#### 2.1.4. Applications.

Arterial network models provide high-resolution arterial pressure and flow waveforms throughout the arterial domain in a fully defined setting. The use of models to simulate PWs is complementary to clinical studies, and offers many advantages: data can be obtained under a wide range of simulated CV conditions; they allow for studying the effect of changes in the model parameters on the wave shape; they are free of measurement error; they can be obtained simultaneously at all measurement sites; they are relatively inexpensive to obtain; and the reference physiological parameters can be specified precisely (105). Consequently, 1-D models have found several applications in CV research. They are valuable tools with which to *1*) study the impact of aging on aortic hemodynamics and wave dynamics (100, 106), *2*) provide mechanistic insights into arterial physiology, pathophysiology, and hemodynamic phenotypes (see Refs. 28, 107–109 for some examples), and *3*) assess the validity of methods and medical devices for the assessment and treatment of vascular aging.

One-dimensional models have been used to assess the validity of methods to estimate arterial system properties (250, 335), methods for pulse wave velocity (PWV) estimation (85, 112–114), estimation of cardiac output (28, 115, 116), estimation of central blood pressure from peripheral pressure (104, 117–119), to detect aneurysms (81, 82, 120) or stenoses (120), or to estimate ventricular contractility (121). Arterial network models have also provided important insights into the performance of loop-based methods for estimating local PWV, demonstrating their susceptibility to the presence of wave reflections (122, 123). The models have been at the basis of a debate on the accuracy of the Arteriograph, a device intended to estimate aortic PWV from a brachial cuff recording inflated to suprasystolic pressures. Model simulations indicated that the device measures brachioaxillary PWV, rather than aortic PWV, because of reflections and re-reflections in the brachioaxillary arterial segment (124). The models have also been instrumental in the debate on the reservoir-wave concept (125), demonstrating inconsistencies in the original formulation of the paradigm leading to spurious interpretation of wave dynamics (and, hence, arterial physiology) confirmed by in vivo experiments (126).

One-dimensional arterial network models may also be particularly suitable to assess the impact of vascular surgical or transcatheter interventions if variables of interest are pressure and flow. Models have, for instance, been used to assess the impact of lower-limb bypass surgery (97, 127) and the creation of a forearm vascular access (arteriovenous shunt) for dialysis on arterial hemodynamics (95). The forearm model was further extended to account for vascular remodeling and was validated in patients, demonstrating its ability to successfully predict maturation of the arteriovenous fistula in patients (128). Another application of 1-D models is the hemodynamic impact of (aortic) (stent) grafts in the arterial tree, which can be described by changing the local stiffness parameter of the desired section in the arterial tree (129). One-dimensional models can only assess the impact of a stent graft on pressure and flow dynamics, and cannot provide any information on the impact of a stent graft on arterial wall stresses or the local flow field.

Nonetheless, the utility and validity of 1-D models should not be overstretched, as computational models have inherent simplifications and assumptions (see *Section 2.1.1*). Most models lack important physiological feedback and control mechanisms, and model results depend on the particular topological network that is being simulated, its boundary conditions, and solution methods. It is not guaranteed that, because a method works on simulated data, it will be applicable in any in vivo setting, where measurement error and biological and physiological variability apply. Conversely, it is reasonable to assume that a method that does not perform well on synthetic data would not perform well in vivo.

### 2.2. 0-D Models

Zero-dimensional models further reduce reality to mathematical descriptions without spacial dimensions. Often these are called lumped parameter models since distributed CV parameters are grouped into single parameters; e.g., distributed vessel elasticity is lumped into vessel compliance, which in turn can be described as a single compliance for the whole arterial tree. For a historical overview on 0-D models see Parker et al. (7) and Westerhof et al. (130). This section focuses on the 0-D formulation (*Section 2.2.1*), the Windkessel (*Section 2.2.2*), and 0-D heart (*Section 2.2.3*) models, and their applications (*Section 2.2.4*).

#### 2.2.1. Formulation.

Zero-dimensional models are described by ordinary differential equations, with time as the only independent variable. The linear 0-D equations for *1*) blood flow in a blood vessel, *2*) the entire arterial tree, or *3*) a portion of it can be obtained from the nonlinear 1-D equations, as described in Technical Supplemental Section 2.2.1. Linearization of the 1-D equations (see Technical Supplemental Section 2.2.1.1) and integration over the vessel length (so that the axial coordinate is eliminated; see Technical Supplemental Section 2.2.1.2) yield the 0-D equations (*Eq. 63*) for blood flow in a vessel segment. These equations are analogous to the transmission line equations and, hence, 0-D models are usually represented by electrical circuits (130) (Fig. 3*B*). Blood flow and pressure are analogous to electric current and potential, respectively. The compliance of the vessel is equivalent to a capacitance; the inertia of blood is comparable with an inductance; and the resistance to blood flow is matched by a resistor. Electrical analog models have been created to simulate blood flow in the systemic arterial tree (132, 133).

By combining the 0-D model equations for each segment of the arterial tree we can obtain a differential equation (see Technical Supplemental Section 2.2.1.3, *Eq. 76*) relating arterial blood pressure in the entire arterial tree to the following CV parameters that are affected by vascular aging (as described in Table 3): time-varying aortic inflow, outflows to the microcirculation, outflow pressure, and distributed physical properties of the vasculature (length, diameter, and stiffness for each arterial segment, and peripheral compliances and resistances). This equation can be solved analytically for blood pressure as a time-varying, space-independent analytical function (*Eq. 80*). It shows the ability of 0-D modeling to approximate distributed 1-D model pressures, particularly during diastole (Fig. 2*B*), and identifies three key factors that describe arterial blood flow: total vascular resistance, total arterial compliance, and outflow pressure. Furthermore, changes in pressure can be assumed to occur synchronously throughout the arterial tree during diastole, with fluid inertia having a negligible effect compared with compliance and resistance (134).

#### 2.2.2. The Windkessel model.

The 0-D model for the arterial tree described by *Eq. 76* leads to the well-known Frank’s two-element Windkessel model (*Eq. 79*) (135) when all peripheral compliances are neglected. The Windkessel model describes the whole arterial tree as a reservoir of constant compliance into which blood flows from the left ventricle (Fig. 3*A*). The time-varying pressure in the reservoir encounters a constant peripheral vascular resistance and flows out into the vascular beds that are at a constant pressure (usually assumed to be right atrial pressure or zero). Despite its simplicity, this model is able to predict the exponential decay of pressure in diastole and the increases in mean arterial pressure and PP with, respectively, increasing resistance and decreasing compliance (Fig. 3*C*); both characteristics of vascular aging. The model predicts a pressure decay with a time constant given by the product of the total resistance and compliance of the arterial network (*Eq. 83*). Hence, it can describe the steeper diastolic pressure decay observed with vascular aging as a result of the smaller exponential time constant produced by the reduction in arterial compliance (Fig. 2*D*). Windkessel models are common choices for outflow boundary conditions in 1-D (see Table 2) and 3-D modeling. They can contain more than two elements and physically exist as bench hydraulic models (Fig. 3*B*) (136).

#### 2.2.3. Heart models and elastance.

There are several possibilities to describe the filing and contraction of the heart in 1-D and 0-D modeling. The simplest approach is to simulate the left ventricle as a “pressure source” and prescribe the pressure PW at the aortic root independently of vascular load. Then, pressure and load together determine blood flow (see *Eq. 82*). Otherwise, the left ventricle can be modeled as a “flow source:” blood flow is forced into the vascular system and the pressure build-up has no impact on the flow. In reality, the heart is neither a pressure source, nor a flow source; i.e., the pressure that the ventricle experiences while ejecting has an effect on the flow it outputs, and vice versa. However, it has been proposed that a hypertrophied heart resembles a flow source; i.e., it can generate output even if the afterload pressure is high. And a failing heart is closer to a pressure source; i.e., pressure may still be maintained but the flow becomes lessened by the load (137).

More physiologically accurate heart models are based on the pressure-volume description of cardiac function (138–140). Ventricular pressure (*P*) plotted versus ventricular volume (*V*) for a complete cardiac cycle produces the so-called PV loop (Fig. 3*E*) (141). This has four phases: *1*) filling phase in diastole (*V* increases with little *P* elevation), *2*) isovolumic contraction phase (no changes in *V*, steep *P* increase), *3*) ejection phase (*V* decreased by stroke volume, relatively moderate *P* alterations), and *4*) isovolumic relaxation phase (no *V* changes, steep *P* drop). During these phases, the ventricle changes from a high-compliance chamber, receiving blood volume with limited *P* increase, to a chamber in which *P* is increased to the extent that it becomes higher than aortic pressure and ejection starts. These phases are conveniently described by the slope of the line defined by a point on the *PV* loop and a fixed point on the volume axis (i.e., the hypothetical *V* when *P* = 0). This slope is referred to as elastance. It measures the rate of change in *P* with the change in *V* and is therefore the reciprocal of compliance (*Section 3.2.4*). It is low in diastole, increases during contraction, and decreases again with relaxation. The end-systolic elastance (*E*_es_) is a measure of ventricular contractility, whereas the end-diastolic elastance is a measure of diastolic myocardial stiffness (70). The slope of the line joining the end-systolic and end-diastolic points in the *PV* loop, called effective arterial elastance (*E*_a_), is a measure of arterial load (142). The ratio *E*_a_*/E*_es_ is a measure of arterial ventricular coupling (75). Table 3 shows the effect of aging on elastance properties. Zero-dimensional heart models often use a time-dependent elastance curve, which is similar for healthy hearts and several heart diseases when normalized by height and peak onset time. As a result, the same curve shape can be used by adjusting the peak based on heart function and heart rate (139, 140).

#### 2.2.4. Applications.

Zero-dimensional models have been used, e.g., to study the load on the heart (afterload) (130), provide mechanistic insights into arterial physiology, pathophysiology, and hemodynamic phenotypes (143–146), estimate central blood pressure from aortic flow (104), and estimate cardiac output (147, 148).

The Windkessel model allows us to describe the Windkessel effect, an important vascular function whereby the pulsatile nature of blood flow is smoothed by the elasticity of the arterial wall. The Windkessel^4^ was used to “store” pressure in a fire engine (Fig. 3*A*). By pumping water into the engine at a higher rate than the flow leaves the spout (where the resistance is located), the pressure in the Windkessel (the compliant chamber) increases, compressing the air inside. As a result, a relatively constant pressure can build up, maintaining flow between the strokes of the pump. This results in less wastage of water that would otherwise drop to the ground between the strokes. In a similar way, the beating heart pumps blood into the compliant large arteries, and since the outflow is restricted by vascular resistance (Fig. 3*A*), blood pressure is built to mean arterial pressure over several cycles. Blood pressure still fluctuates between diastolic and systolic values (Fig. 3*C*), but not between systolic and nearly zero pressure, as it does within the ventricle (Fig. 3*E*). Thus, the pressure drop in diastole is limited, the PP is reduced, and the flow is continued between heartbeats. When the arteries become stiffer with aging and disease, the Windkessel function is decreased because of the decrease in compliance and, hence, PP increases (Fig. 3*C*), in agreement with in vivo measurements (149, 150).

Closed-loop 0-D models have been created to describe the entire circulation using *1*) lumped-parameter resistors, capacitors, and inductances to simulate blood flow in the arterial and venous vasculatures and *2*) elastance functions to model the right and left heart chambers (151–153). The vasculature may be divided into several compartments representing, for example, thoracic, abdominal, and more distal vasculatures. Closed-loop models are used to study cardiac and vascular pathophysiology for the whole CV system in the neonate, children, and adult (5, 154, 155) and the effect of aging (156). Changes or redistribution of blood volume (e.g., by a changed unstressed volume of the veins) can be modeled in a straightforward manner, as well as the effects of autonomic control of the heart and vasculature (138, 151, 157).

## 3. PULSE WAVE ANALYSIS METHODS

This section reviews methods for analyzing PW signals and extracting relevant information for vascular age assessment. It begins with considerations of how to obtain in vivo PW signals (*Section 3.1*) that are ready to be analyzed using theoretical-based (*Section 3.2*) and empirical-based (*Section 3.3*) methods. Both types of analysis techniques can also be applied to in silico PW signals, which often do not require the preprocessing steps described in *Section 3.1.2* since they are free of measurement errors and artifacts affecting their quality.

### 3.1. In Vivo Pulse Wave Signals

The arterial PW can be represented by blood pressure, flow or velocity, arterial distension, and PPG signals. These are continuous signals, showing a typical repeating pattern with each heartbeat. The characteristics and morphologies of PW signals, which differ between different types, measurement sites, and ages, need to be considered when measuring (*Section 3.1.1*) and preprocessing (*Section 3.1.2*) them.

#### 3.1.1. Measurements.

PW signals can be probed centrally (e.g., heart, aorta, carotid arteries) or on peripherally accessible measuring sites (e.g., upper arm, wrist, groin, finger, toe, retina, and earlobe). Peripheral PWs differ from central PWs because of the effects of PW propagation and reflection along the arterial tree. Signals can be directly measured with methods such as pressure catheters, applanation tonometry, volume clamp method, oscillometric cuff (179, 180), (Doppler) ultrasound (336), and magnetic resonance imaging (MRI) (181), but are also increasingly accessible to wearable technologies such as photoplethysmography (182), which is acquired by pulse oximeters and consumer devices (e.g., smartwatches and smartphones). These techniques are compared in Table 5 in terms of characteristics of the PW signals they measure and preferred features for measuring the PW in daily life. Photoplethysmography is widely used because of its ease of use; however, the PW signal it measures is not as well understood physiologically as the signals measured by the other techniques. Pressure catheters are the gold standard for measuring blood pressure and central hemodynamics; however, they are an invasive technique, making them unsuitable for large studies and used in apparently healthy individuals. Ultrasound and MRI provide noninvasive and accurate measurements of blood velocity, blood flow, and luminal diameter, tonometry is a practical technique for noninvasively measuring arterial pressure waveforms, and the use of cuffs to measure PW has become increasingly popular recently. These techniques have been used in many large epidemiological studies and in routine clinical practice, but they are generally not suitable for self-measurement since most require specialist equipment and trained operators.

**Table 5.** Comparison of pulse wave measurement techniques

	Pressure Catheter	Applanation Tonometry	Volume Clamp	Oscillometric Cuff	PPG	US	MRI
Signal type	*P*	*P*	*P*	*P*	PW	*U*, *Q, D*	*U*, *Q*, *D*
Central signal	+	+	−	−	−	+	+
Peripheral signal	+	+	+	+	+	+	+
Noninvasive	−	+	+	+	+	+	+
Continuous acquisition	+	−	+	−	+	−	−
No trained operator	−	−	−	+	+	−	−
Calibrated	+	−	+	+	−	+	+
Unobtrusive monitoring	−	−	−	+	+	−	−

The first three rows show characteristics of the pulse wave signals directly measured by each technique, and the remaining rows compare techniques in terms of preferred features for measuring the pulse wave in daily life. *D*, luminal diameter; MRI, magnetic resonance imaging; *P*, blood pressure; PPG, photoplethysmography; PW, pulse wave signal with arbitrary units; *Q*, blood flow rate; *U*, blood flow velocity; US, ultrasound.

Since arterial PW analysis depends on detailed features of PW morphology, the reliability of the measurements is crucial. Measurement methods are sensitive to technical errors [e.g., damping or ringing of the catheter-manometer system (183, 184)], artifacts [e.g., movement during MRI acquisition (185)], operator-dependent inaccuracies [e.g., an incorrect insonation angle for Doppler measurements (186)], and physiological effects [e.g., respiration induced changes (187)]. The frequencies of interest in the PW signal are below 20 Hz for adults (188, 189), which means that a sampling frequency of at least 40 Hz is needed according to the Nyquist theorem (190). In general, the greatest care should be taken to obtain good quality data, since in most cases it is impossible to correct measurements afterward.

#### 3.1.2. Preprocessing.

A PW signal is typically preprocessed before analysis to improve the reliability of the analysis. The process consists of several steps, which are summarized in Fig. 4. First, individual PWs are identified for analysis using a beat detection algorithm (193, 194). Second, any periods of signal that are incomplete, of low quality, or that contain data outside of the plausible measurement scope should be excluded. Techniques for assessing the quality of blood pressure and PPG signals are reviewed in Refs. 195 and 196. Third, high-frequency noise (e.g., electrical interference) and low-frequency variations (e.g., due to respiration) can be eliminated through digital filtering (61, 196). The filter design is important: different filter cutoff frequencies may be required for different analyses (197); and zero-phase digital filtering should be used to prevent any phase shifting (196). After filtering, a sufficient number of harmonics should still be present in the signal since excessive filtering can lead to the loss of important information in the PW shape.

There are several additional preprocessing steps that can optionally be performed, as illustrated in Fig. 4: *1*) calibrating blood pressure PWs acquired by applanation tonometry with an independent pressure measurement to convert the piezoelectric measurement (voltage) into a pressure signal (198); *2*) transforming a peripheral PW to a central PW using a transfer function (117, 199); and *3*) ensemble averaging PWs to create a single, averaged PW (169). Other considerations include *4*) interpolating signals to increase their temporal resolution, which is particularly helpful when extracting the timings of PWs (such as for PWV estimation) (200); *5*) calculating derivatives for PW analysis [e.g., using Savitzky–Golay filtering (201)]; and *6*) when working with multiple simultaneous PW signals, ensuring that they are time aligned, such as by the times of systolic upstrokes (see *Section 3.3.1*), by waveform matching, or by cross correlation (202).

### 3.2. Theoretical-Based Analysis Methods

The 1-D and 0-D blood flow models presented in *Section 2* form the basis of several theoretical PW analysis methods used to assess vascular age. These methods offer valuable insights into the interpretation of PW morphology and its relationship to CV parameters associated with vascular aging and are subsequently reviewed. Table 6 shows how vascular age indices obtained using these methods vary with chronological age, accounting for sex differences if available.

**Table 6.** Evolution of vascular age indices with chronological age

Index	Age Variation	Study Type	References
Forward pressure wave amplitude	Inc. in M/F	C	(158)
	Dec. in M/F	L	(159)
Backward pressure wave amplitude	Inc. initially in M/F; flattening in M and falling in F	C	(158)
	Dec. in M/F	L	(159)
Wave intensity	Dec. in forward compression wave in M/F; inc. in backward compression wave in M/F; no change in forward decompression wave in M/F	C	(160–163)
Local pulse wave velocity	Inc. in M/F	C	(161, 162)
Regional pulse wave velocity	Exponential inc. in M/F; minor inc. in childhood	C	(3, 78, 164)
	Exponential inc. in M/F (F > M)	L†	(3, 159)
Compliance	Inc. in M/F up to 30 yr old; dec. after 50 yr old (F > M)	C	(165, 166)
	Inc. in 35–40 yr old M (but not in F); minor dec. over 40–55 yr old in M/F	L	(159)
Aortic input impedance	Inc. in M/F; rightward shift of the minimum modulus	C	(166, 167)
	No significant changes over 10 yr in middle-aged M/F	L	(159)
Aortic characteristic impedance	No significant change	C	(168)
Pulse pressure	Exponential inc. in M/F; minor inc. (or dic.) in childhood	C	(3, 164)
	Exponential inc. in M/F; can dec. in young and elderly M	L	(3)
Pulse pressure amplification	Dec. in M/F	C	(164, 169)
Augmentation index	Inc. in M/F (M > F); dec. in childhood	C	(3, 164)
Augmentation pressure	Linear inc. in M/F (F > M); plateau in elderly M	C	(3, 164)
Ankle-brachial index	Inc. in M/F up to 60–69 yr old; dec. after (M > F)	C	(170)
Ambulatory arterial stiffness index	Inc. in M/F (F > M)	C	(171)
Cardio-ankle vascular index	Linear inc. in M/F (M > F)	C	(172)
Flow augmentation index	Quadratic upwardly concave inc. in M/F	C	(173)
Reverse-to-forward flow index	Inc. in M/F (aorta); no significant change (femoral artery)	C	(174, 175)
Diastolic-to-systolic forward flow ratio	Dec. in M/F (femoral artery)	C	(174)
Photoplethysmogram second derivative	Linear inc. in *b*/*a* and aging index in M/F; linear dec. in *c*/*a*, *d*/*a* and *e*/*a* in M/F	C	(176–178)

These indices have been calculated using the theoretical-based and empirical-based methods described in *Sections 3.2* and *3.3*, respectively, in cross-sectional (C) or longitudinal (L) studies. Variations with chronological age are given for adult males (M) and females (F), and in childhood if available. dec., decrease; inc., increase.

†Results found for carotid-femoral and brachial-ankle pulse wave velocity only.

#### 3.2.1. Forward- and backward-traveling waveforms.

**Figure 5.**
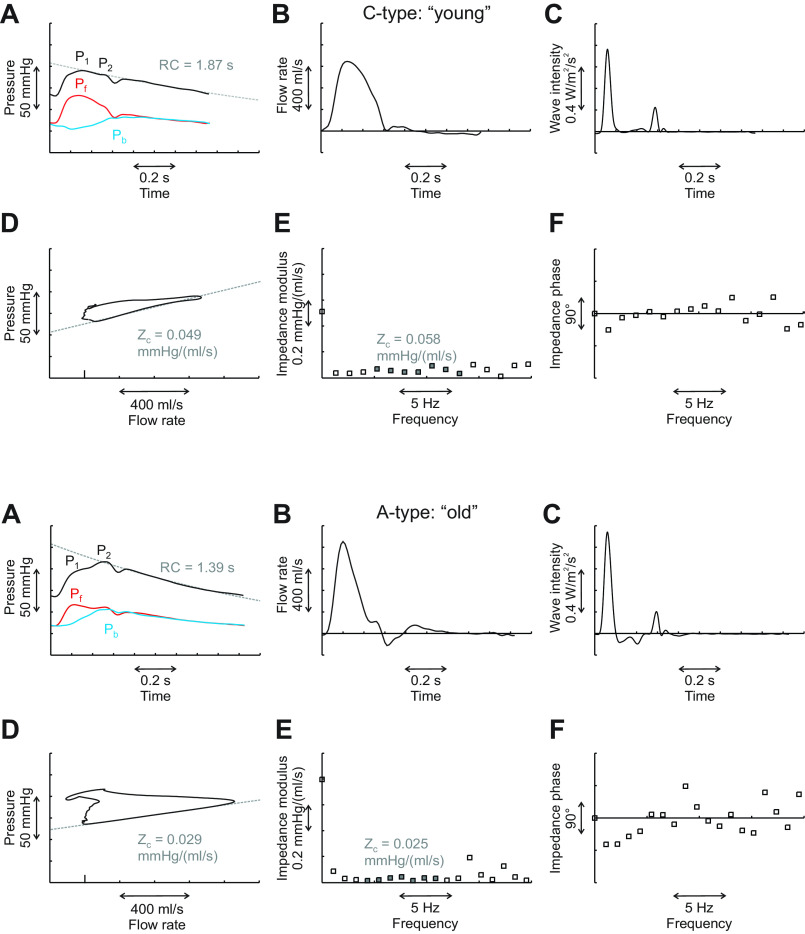
Theoretical-based methods of pulse wave (PW) analysis. The following methods are applied to ensemble averaged pressure and flow waveforms measured in the ascending aorta of young (*top*) and old (*bottom*) subjects (149). *A*: pressure wave separation into forward (*P*_f_, red)- and backward (*P*_b_, blue)-traveling components using the flow rate in *B*. RC is the time constant of the total pressure wave (black). P1 and P2 are the inflection points described in *Section 3.3.1*. *C*: wave intensity analysis. *D*: pressure-flow loop with the calculated characteristic impedance (*Z*_c_, see *Eq. 134*) from the straight portion. *E*: impedance modulus. *F*: impedance phase. *E* shows *Z*_c_ calculated from the 4th to 10th harmonics (filled boxes).

Theoretical analysis of the 1-D model governing equations reveals the presence of PW motion in flexible blood vessels (see Technical Supplemental Section 3.2.1.1). PWs propagate in the forward direction (i.e., from the heart to the periphery) and interact with tapered vessels and bifurcations, producing reflected waves that travel back toward the heart, where they can be rereflected into forward-running waves (203). This analysis enables separation of the blood pressure, *P*, and blood flow velocity, *U*, waveforms measured at the same location into forward-traveling (*P*_f_, *U*_f_) and backward-traveling (*P*_b_, *U*_b_) waves (Fig. 5*A*); i.e., *P* = *P*_f_ + *P*_b_ and *U* = *U*_f_ + *U*_b_ (204) (see Technical Supplemental Section 3.2.1.2). The amplitude of *P*_b_ and the ratio of the *P*_b_ amplitude to the *P*_f_ amplitude have been shown to be an independent predictor of CV events (205, 206). Changes in the amplitudes of *P*_f_ and *P*_b_ have been observed with aging (see Table 6).

The separation into forward- and backward-traveling waveforms can identify the direction (forward or backward) of the waves that make up *P* and *U* at a given time within the cardiac cycle. However, this method cannot provide the physical locations in the CV system where the waves originated (207, 208). Alternative separation techniques have been suggested to achieve this when analyzing in silico (208) and in vivo (125, 203) data, although their potential for assessing vascular age and disease has been questioned (209, 210), or needs to be investigated (203).

#### 3.2.2. Wave intensity analysis.

Wave intensity is the rate of energy flux per unit area carried by the PW, and is analogous to acoustic intensity (211). It can be calculated from simultaneous *P* and *U* measurements at any location in the arterial network using *Eq. 101* (Fig. 5*C*) and separated into forward- and backward-traveling components using *Eq. 107*. As shown in Technical Supplemental Section 3.2.2.1, wave intensity measures the prominence of changes in *P* and *U* in the forward and backward directions at any time during the cardiac cycle (204, 211). It is particularly well suited to understanding the role of wave reflections on pressure and flow in systemic arteries (212–219), including coronary circulation (220–222). In vivo studies at the ascending aorta have shown that the magnitude and arrival time of the backward compression wave in mid-systole varies with age (see Table 6), disease, arterial compliance, and vascular tone (213, 223), and the magnitude of the forward compression wave is a predictor of cognitive decline (160).

The units of wave intensity vary between studies, which can limit comparison of data across different studies (see Technical Supplemental Section 3.2.2.2). In addition, several other factors affect absolute wave amplitude. Noise reduction, using techniques described in *Section 3.1.2*, is crucial in reducing the effect of noise when calculating wave intensity (224). Moreover, time alignment of *P* and *U* is critical for accurate calculation of wave intensity, since misalignment can lead to inaccurate wave profiles and artifactual waves. Finally, differentiating between “real” wave peaks and background noise can be challenging, but may be aided by a recently described maximum entropy technique (225).

In an attempt to use wave intensity analysis as a diagnostic tool, the equations can be rewritten using luminal diameter and flow velocity (see Technical Supplemental Section 3.2.2.3); both of which can be measured noninvasively (see Table 5). In healthy subjects, this noninvasive wave intensity has shown a decline in left ventricular early and late systolic functions with age (161) and a greater effect of the aging process on the carotid than the femoral artery (162).

Numerical studies using 1-D blood flow modeling have shown that wave intensity can identify the timing, direction, and magnitude of the predominant waves that shape aortic pressure and flow waveforms in systole. However, wave intensity fails to identify the important contribution of wave reflections during diastole and those arising from pulses in previous cardiac cycles (209, 226, 227). This occurs because wave intensity analysis tends to accentuate high-frequency waves, whereas repeated reflections in the arterial tree, along with wave dispersion, attenuate high frequencies and lead to low-frequency waves predominating during diastole (227, 228).

#### 3.2.3. Pulse-wave velocity.

PWV is the speed by which the PW travels in arteries. Several methods have been proposed to determine PWV, which can broadly be grouped under two categories based on *1*) local and *2*) regional measurements. Local methods use measurements at a single location of either pressure and velocity, diameter and velocity, flow rate and area, or pressure and diameter. The classical 19th-century Moens-Korteweg equation (*Eq. 116*) (229, 230) was originally introduced in the context of flows in thin elastic tubes. It relates local PWV to the geometrical and mechanical properties of the local arterial wall, showing an increase in PWV with increasing elastic modulus (i.e., wall stiffness) and wall thickness, and with decreasing luminal radius. Therefore, PWV quantifies arterial stiffness, which is of clinical interest for assessing the arteriosclerosis component of vascular aging (3). In the 20th century, the Bramwell–Hill equation was introduced, which describes the relationship between local PWV and local arterial wall distensibility (*Eq. 111*). In Technical Supplemental Section 3.2.3.1, the Bramwell–Hill equation is derived from the 1-D model governing equations. In Technical Supplemental Section 3.2.3.2, the Moens–Korteweg equation is derived from the Bramwell–Hill equation.

Early in the 21st century, a series of techniques for estimating local PWV were introduced. These can be classified as loop and sum-of-squares methods. Most of the loop methods rely on the existence of a reflection-free period within the cardiac cycle (231–234) (Fig. 5*D*) (see Technical Supplemental Section 3.2.3.3). The sum-of-squares technique (Technical Supplemental Section 3.2.3.4) was introduced to assess local PWV from simultaneous pressure and velocity measurements in the coronary arteries, where a reflection-free period cannot be safely assumed during the cardiac cycle (235). These methods have primarily been studied using in vitro and in silico data. Novel derivations of the sum-of-squares method for diameter and velocity, or flow rate and area are provided in Technical Supplemental Section 3.2.3.4.

Regional methods require pulse waveforms measured at two arterial sites; e.g., along the aorta (236, 237), at the carotid and femoral arteries (238), or at the brachial and ankle arteries (239). Regional PWV is calculated as the ratio of the distance between the two measurement sites to the time delay for the wave to travel from one site to the other. Distances are typically measured from surface markings or intra-arterial distance, and time delays by identifying the feet of the two waves measured at the two locations or using cross-correlation methods (113, 240). Regional PWV measures increase with age starting in childhood (see Table 6). The effects of aging, however, are not uniform in systemic arteries: central arteries such as the aorta stiffen with age more than peripheral arteries in the arms and legs (241). Regional PWV measures have been combined with CV risk factors (242) and a measure of atherosclerosis, such as coronary artery calcification (243), intima-media thickness (244), to quantify an individual’s vascular age. Alternatively, regional PWV alone may be able to quantify an individual’s vascular age (243) and predict CV events and mortality (245, 246).

Computational blood flow modeling has been used to assess the performance of local (122, 226, 234, 247) and regional (85, 113, 124) measures of PWV. These studies have shown that *1*) methods using aortic PW data as well as the carotid-femoral foot-to-foot method are accurate indicators of aortic stiffness, *2*) other local and regional methods tend to over- or underestimate aortic PWV, and *3*) large PW reflections have an adverse effect on the accuracy of PWV estimates.

#### 3.2.4. Compliance.

Compliance is the rate of change in blood volume with the change in blood pressure (often expressed in mL/mmHg or m^3^/Pa). It quantifies the buffer capacity or Windkessel effect of the vasculature (see *Section 2.2.4*) and is directly related to arterial size and inversely related to local PWV (see Technical Supplemental Section 3.2.4). Direct measurement of compliance is impossible, as it would require sealing off the arterial tree for a pressure inflation test. The most simple estimate of compliance is the ratio of stroke volume to PP (248), but this method does not account for arterial outflow in systole and overestimates compliance (249).

Other methods implicitly or explicitly rely on an assumed Windkessel model (Fig. 3*B*) (130). In the decay time method, an exponential is fitted to the diastolic part of the aortic pressure wave, with the time constant providing an estimate of the product of total peripheral resistance and compliance, also termed the decay time of the arterial system (250) (see *Eq. 83* and Fig. 5*A*). A variation of this method is the area method (251), computing the decay time from the area under the diastolic pressure waveform, rather than fitting an exponential. Note that to obtain compliance using methods that estimate the decay time, resistance must be calculated from mean arterial pressure and flow (cardiac output). To eliminate the sensitivity of the area and decay time method to wave morphology (that may be far from exponential) or the selected diastolic segment, the PP method was introduced (111, 252): an iterative method estimating compliance through minimizing the difference between the measured PP and the pulse predicted by a two-element Windkessel model. The method is robust with results highly correlating with the ratio of stroke volume to PP (249). Virtually all other compliance estimation methods make use of more complex Windkessel models consisting of more elements (3-element or 4-element Windkessel models) (110, 251, 253) and nonlinear terms, including pressure-dependent (254) or frequency-dependent compliance (255) (see Technical Supplemental Section 3.2.4).

With compliance depending on both arterial stiffness (which is dependent on blood pressure) and arterial size, the relation with age is not straightforward. It has been reported to increase up to age 30, vary little in middle age, and decline rapidly above age 50 (165). Longitudinal analysis of data from round 1 of the Asklepios study in subjects aged 35–55 yr confirmed a relatively constant compliance in males, but a decrease in compliance in females (168), consistent with the study by Waddell et al. (166). Longitudinal data from the Asklepios population, with an effective age change of ∼10 yr, showed an increase in compliance in the younger men (35–40 yr at baseline) but not in females. Compliance remained fairly constant at the higher age categories, suggesting that the increase in stiffness (increase in PWV) is balanced by a change in aortic dimensions in this age range (159).

#### 3.2.5. Input impedance and characteristic impedance.

Although hydraulic resistance is calculated as the ratio of mean pressure drop (difference between inlet and outlet of the resistance) and mean flow, impedance can be defined as the ratio of the pulsatile components of pressure and flow. Rather than viscous friction, inertia of the blood and vessel stiffness are the determinants of impedance (256). The terminology is adopted from electrical engineering. Impedance is typically calculated in the frequency domain, after Fourier decomposition of pressure and flow into harmonics (see Technical Supplemental Section 3.2.5). It is a complex number, most often represented in a modulus (Fig. 5*E*) and phase (Fig. 5*F*) notation, where the modulus represents the ratio of the amplitude of corresponding pressure and flow harmonics, and the phase angle the phase delay between both (see *Eq. 135*). When calculated from ascending aorta pressure and flow, it is termed input impedance (often denoted as *Z*_in_) and constitutes a global systemic description that characterizes the cumulative effect of wave travel and reflection from the arterial tree and constitutes the afterload of the heart (257, 258).

A special case arises for a system that is free of reflections. *Z*_in_ is reduced to characteristic impedance (*Z*_c_), which can be shown to approximate the ratio of the product of blood density and local PWV to the vessel cross-sectional area (*Eq. 137*). Therefore, *Z*_c_ constitutes a local arterial parameter. Since, for high frequencies, the arterial system can be considered to be reflectionless because of destructive interference of reflected waves (259), *Z*_in_ at high frequencies approaches *Z*_c_ (*Eq. 146*). Hence, *Z*_c_ can be calculated by averaging the modulus of *Z*_in_ between the fourth and tenth harmonics (Fig. 5*E*) (260). Alternatively, *Z*_c_ can also be estimated in the time domain from the ratio of changes in pressure and flow in early systole (Fig. 5*D*) (261) (see Technical Supplemental Section 3.2.5).

Arterial impedance has been used less often to assess vascular age. Aortic *Z*_in_ has been shown to increase with aging in healthy populations (167), suggesting that it may constitute a relevant indicator of age-related CV risk. A study involving over 2,000 healthy individuals aged 35 to 55 found *Z*_in_ to evolve from a pattern indicative of wave transmission and reflection in the younger to a pattern more compatible with a Windkessel-like system in the elder. In women, but not in men, a decrease in total arterial compliance led to an increased *Z*_in_ in the low-frequency range. Little to no changes with age were observed in *Z*_c_, possibly because of compensatory effects of aortic dilatation and stiffening (168). Albeit, arterial impedance can provide major mechanistic insights into age-related changes in vascular function, other parameters (e.g., PWV) are required for a more complete interpretation and to disentangle the effects of changes in stiffness from changes in arterial dimensions.

### 3.3. Empirical-Based Analysis Methods

**Figure 6.**
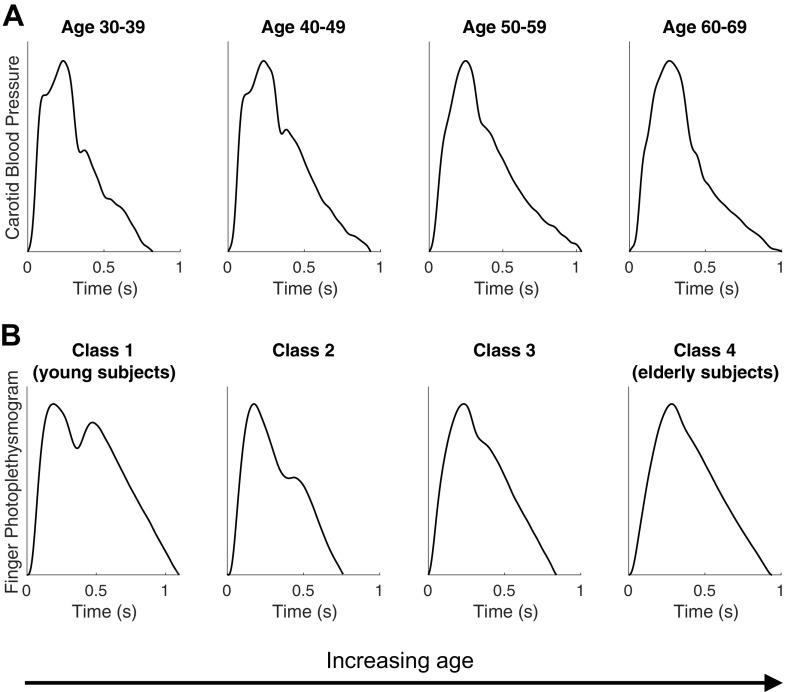
Changes in in vivo pulse waves (PWs) with age: changes in carotid pressure PWs (*A*) and changes in finger photoplethysmogram (PPG) PWs (*B*), labeled with classes as it is common for this type of PW (269). Sources: blood pressure data from the Asklepios data set (334); photoplethysmogram data from the VORTAL data set (182). Figure adapted from “Classes of photoplethysmogram (PPG) pulse wave shape,” Wikimedia Commons, under CC-BY 4.0.

This section reviews indices of vascular age that are based on empirical analyses of PW morphology or on semiempirical analyses incorporating theoretical concepts described in *Section 2*. These indices require the identification of fiducial points on a PW (*Section 3.3.1*) from which indices of vascular age can be calculated (*Section 3.3.2*). Figure 6 shows examples of how the shapes of *1*) carotid blood pressure and *2*) finger PPG PWs change with age, allowing the effects of aging to be elucidated from the shapes of PWs.

#### 3.3.1. Fiducial points.

**Figure 7.**
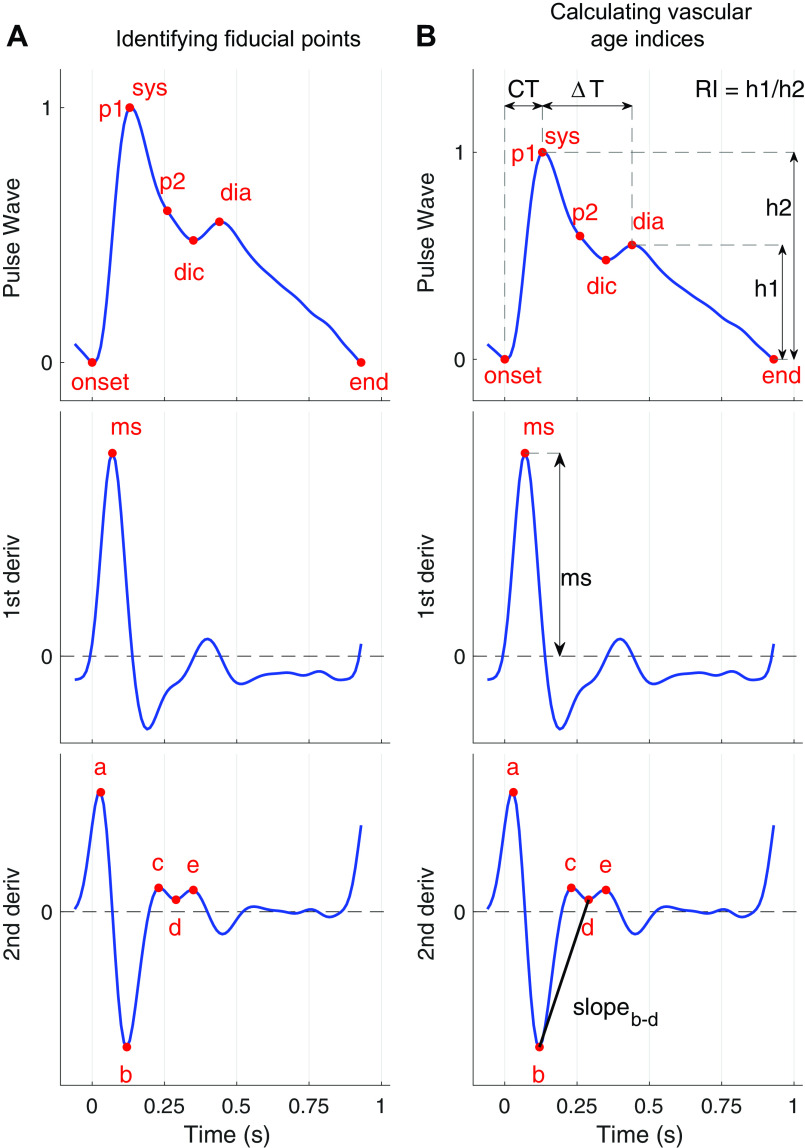
Extracting vascular age indices: vascular age indices can be obtained from a single photoplethysmogram (PPG) pulse wave (PW) in two steps. *A*: identifying fiducial points on the PW [systolic (sys) and diastolic (dia) peaks, dicrotic notch (dic), early and late systolic peaks (*p*1 and *p*2)], its first derivative [slope of the rising front (ms)], and its second derivative (*a*, *c*, *e* peaks and *b* and *d* troughs). *B*: calculating features from the amplitudes and timings of these points, such as the time from pulse onset to sys (CT), the time from sys to dia (Δ*T*), the reflection index (RI), the maximum upslope (ms), and the slope between *b* and *d* troughs (slopes *b–d*). Sources for *A* and *B*: Peter Charlton, Photoplethysmogram (PPG) pulse wave fiducial points (CC tBY 4.0; *A*) and Photoplethysmogram (PPG) pulse wave indices (CC BY 4.0; *B*) https://commons.wikimedia.org/wiki/File:Photoplethysmogram_(PPG)_pulse_wave_indices.svg.

Several fiducial points can be identified on a PW signal, as shown for the PPG PW in Fig. 7*A*. Accurate identification of these fiducial points is crucial for reliable analysis of PW shape. We now describe the methodology for identifying fiducial points that are common to all PW types, followed by the methodology for fiducial points that are specific to different types of PWs.

##### 3.3.1.1. Systolic and diastolic phases.

**Figure 8.**
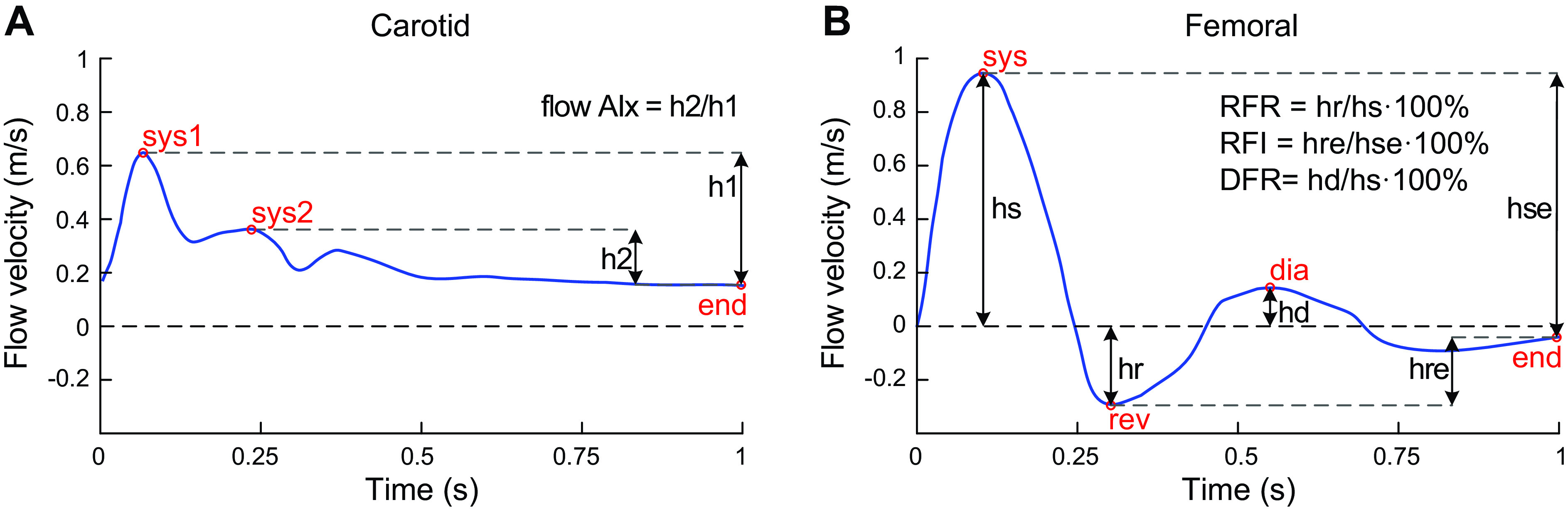
Definition of fiducial points and indices on flow velocity pulse waves (PWs). *A*: flow augmentation index (AIx) can be obtained, e.g., in the carotid artery, using fiducial points of early (sys1) and late (sys2) systolic peak velocities, and end-diastolic (end) velocity. *B*: on bidirectional PWs, e.g., in the femoral artery, reverse-to-forward flow ratio (RFR), reverse-to-forward flow index (RFI), and diastolic-to-systolic forward flow ratio (DFR) can be obtained using fiducial points of systolic (sys) and diastolic (dia) forward peak velocities, and reverse (rev) peak velocity. Sources: flow data taken from Refs. 173 and 174.

PWs can be separated into systolic and diastolic phases, where the systolic phase corresponds to the time during which blood is ejected from the left ventricle into the aorta, and the diastolic phase corresponds to the time during which no blood is ejected. When PWs are measured at sites close to the heart, the end of systole can be identified on blood pressure waves as the time of the dicrotic notch (as described later), and on flow velocity PWs as the time at which the flow velocity reduces to (close to) zero. Under normal physiological conditions, flow into the aorta is zero during diastole (Fig. 5*B*), but more distally a positive diastolic flow may be present (Fig. 8).

##### 3.3.1.2. Pulse onset.

The pulse onset is the local minimum at the start of each PW, often called the foot of the wave, which indicates the beginning of systole (see red dots in Fig. 4*A*). The simplest approach to identify the pulse onset is as the point corresponding to the minimum value on a PW. On pressure PWs this corresponds to the diastolic blood pressure (DBP). Several more complex approaches have been proposed to more accurately identify the pulse onset for use in pulse transit time measurement (113). For example, the intersecting tangents method fits a line to the upstroke and another line to the preceding diastolic decay (or simply a horizontal line to the diastolic minimum). The intersection of the two tangents is then used to define the onset of the wave. Other approaches include using the maximum in the first or second derivative (202), or using a slope sum function to accentuate the systolic upstroke and therefore more clearly define the pulse onset (262). The choice of approach can influence the accuracy of parameters derived from the timing of the pulse onset, such as PWV (240, 263).

##### 3.3.1.3. Systolic peak.

The systolic peak is the highest point on a PW, separating the systolic upstroke from the systolic downstroke. On pressure PWs this corresponds to systolic blood pressure (SBP) and on flow velocity PWs it corresponds to systolic forward peak velocity (Fig. 8). PPG PWs exhibit a similar systolic peak (Fig. 7, *top*), although the timing of the systolic peak can vary greatly between measurement sites (such as finger and wrist) (264).

##### 3.3.1.4. Dicrotic notch.

In pressure PWs, the closure of the aortic valve causes a (relatively sharp) notch called an incisura. This may be seen at the end of systole in central pressure PWs, at approximately one-third of the heart period, and around one-third down the descending part of the wave (Fig. 5*A* and Fig. 6*A*; ages, 30–39 and 40–49 yr). The notch may be followed by a secondary dicrotic wave because when the aortic valve closes, the elastic recoil of the aorta can cause a small increase in blood pressure (265). As the wave travels away from the heart, the waveform changes with the incisura becoming less sharp, which is then called a dicrotic notch, and also sometimes followed by an increasing secondary dicrotic wave (266) (Fig. 6*A*; ages, 30–39 and 40–49 yr). With the change in the waveform, the time relation of the dicrotic notch and the incisura as functional measures of end ejection may become disturbed.

A range of algorithms has been proposed to identify the dicrotic notch. The first approach is to analyze the original pressure PW, or its first or second derivatives (267). A challenge with this approach is to correctly identify dicrotic notches even when they only manifest as an incisura rather than a notch. An alternative approach is to estimate an arterial flow signal from the pressure signal using a three-element Windkessel model, and then identify the dicrotic notch as the minimum of the first negative dip in the flow signal after the systolic peak (268). Figure 6*A* illustrates some of the difficulties in detecting the dicrotic notch. It is clearly visible on the downslopes of the pressure PWs for 30- to 39- and 40- to 49-yr-old subjects (at ∼0.3 s). However, it manifests as a less clear incisura in the 60- to 69-yr-old PW. Similarly, dicrotic notches can be less clear at more peripheral measurement sites. It has been suggested that the presence or absence of a clearly defined dicrotic notch in a peripheral pressure PW may be indicative of CV health or aging (269).

The PPG signal can exhibit a trough at a similar time to the dicrotic notch on a pressure PW (e.g., the Class 1 wave in Fig. 6*B*). Although this may look similar to the dicrotic notch, it is not yet clear how closely this corresponds to the end of systole (270).

##### 3.3.1.5. Fiducial points on pressure PWs.

The shape of the pressure PW is determined by the shapes of the forward and reflected waves (Fig. 5*A*). The addition of reflected waves to the forward wave may result in an inflection point during systole or anacrotic notches (i.e., notches in the systolic upstroke). An inflection point occurs where the slope of a curve changes from becoming less steep to becoming steeper again (formally: the curvature changes from “concave downward” to “concave upward”). Inflection points are typically identified from higher-order derivatives of the curve. When the inflection point occurs before the systolic peak [an A-type PW as shown in Fig. 5*A*, *bottom* (149)], the pressure at the inflection point is termed P1, and the systolic pressure is termed P2 (271). In this case, P1 can be defined as coincident with the inflection point (identified using the 4^th^ derivative) (272), or alternatively defined as the “shoulder point” just before the inflection point (identified using the 2^nd^ derivative) (273). When the inflection point occurs after the systolic peak [a C-type PW as shown in Fig. 5*A*, *top* (149)], the systolic pressure becomes P1 and the pressure at the inflection point becomes P2. In this case, P2 can be identified using the 2^nd^ derivative (273).

The wave shape changes when traveling from the heart to the periphery and with it, the timing and height of the inflection points and anacrotic notches. The A-type wave commonly seen in the aorta transforms to a C-type in the more distal vessels. A central A-type with a small augmentation (i.e., difference between P1 and P2) is related to a low postsystolic inflection point in the periphery, whereas a high A-type augmentation corresponds to a high peripheral inflection point (271). Since it thus seems that the same information may be obtained from a peripherally and centrally measured wave, it has been suggested that preprocessing by using a transfer function to reconstruct the corresponding central wave is not necessary (274). Extensive derivative-based analysis techniques for peripherally measured waves have been developed, see for instance Ref. 176.

##### 3.3.1.6. Fiducial points on PPG PWs.

The finger PPG signal is typically characterized by multiple points (61). The PW can exhibit a diastolic peak, indicated by “dia” in Fig. 7, *top*. It has been hypothesized that this peak is caused by wave reflections, which is why it is more prominent in younger subjects (Fig. 6*B*) (275). The first derivative is dominated by a point of maximum slope, indicated by “ms” in Fig. 7, *middle*. The second derivative is typically described using five fiducial points, named a, b, c, d, and e, whose amplitudes vary with age (176) (Fig. 7, *bottom*). Algorithms have been proposed to identify these fiducial points (276, 277). Much of the literature on these fiducial points is based on the analysis of finger PPG PWs. Further research is required to determine whether they can be accurately identified at other anatomical sites such as the wrist (264).

##### 3.3.1.7. Fiducial points on flow PWs.

In cases where flow augmentation is present, such as in a common carotid artery, the systolic phase of the flow velocity PW may contain not a single systolic peak, but early and late systolic peaks, which can also be called shoulders (Fig. 8*A*) (173). A bidirectional flow velocity PW, which can be seen, e.g., in the femoral artery (Fig. 8*B*) or the distal aorta, exhibits the reverse peak velocity (174, 175). Since the volume flow rate is assessed from the cross-sectional area of and blood flow velocity at the artery of interest, the flow rate and flow velocity waves exhibit similar morphology (assuming that the influence of arterial diameter is negligibly small).

#### 3.3.2. Indices of vascular age.

Several indices of vascular age can be calculated from fiducial points identified on pressure, flow, and PPG PWs. Table 6 shows the evolution of these indices with aging.

##### 3.3.2.1. Pressure indices.

Pulse pressure (PP), the difference between systolic and diastolic pressures, is an easily accessible measure of vascular age (278) which is associated with unfavorable CV outcomes (279). The increase in PP with aging has several consequences for CV health, including *1*) increased left ventricular afterload leading to left ventricular remodeling, dysfunction, and failure (280, 281) and *2*) microvascular lesions in high-flow/low-resistance organs, such as the brain and kidneys, by increased transmission of pulsatile pressure and flow to the microvasculature (282, 283).

Pulse pressure amplification (PPA) describes the increase in PP along the arterial tree. It is primarily quantified as the ratio of distal to proximal PP (284), although other definitions have been proposed (285, 286). Amplification decreases with age (164, 169), increases with heart rate (287), and is different for men and women (288). A lower PPA is usually associated with increased CV risk (205, 289) and vascular aging (290).

The difference in pressure between P1 and P2 is called augmentation pressure (or Δ*P* in the original publication) (149). Δ*P* indicates the rise in pressure because of reflected waves. It is often expressed as the augmentation index, AIx = Δ*P/*PP. When the inflection point occurs after the systolic peak (C-type waves shown in Fig. 5*A*, *top*), P1 *>* P2, and therefore Δ*P* < 0 and AIx *<* 0: this does not imply that the reflected pressure wave is negative, but that it starts contributing at a late point in time. Δ*P* and AIx were introduced as measures of wave reflection (238), but the relation between the two is not straightforward (see also Refs. 105 and 108). In general, Δ*P* and AIx increase with age (see the A-type waves in Fig. 6) (168, 291). AIx has been proposed as a surrogate for PWV. However, the association between AIx and PWV is limited (292), or even nonexistent when using invasive measurements (293). Although arterial stiffness continues to increase with age, the rise in AIx levels off around the age of 60 yr (164, 294). The increase with age of AIx is related to the decrease in PP amplification (169). Despite the interpretation of AIx not being straightforward, several significant associations have been established between AIx and CV disorders (295–298).

Since ventricular ejection patterns influence the shape of the forward wave (and thus, also of the reflected wave) (203), heart function has an impact on the inflection points from which Δ*P* and AIx are calculated (299). This at least in part explains the obfuscated relation between AIx and timing of the reflected wave (191). Recently, the notion that inflection points are not purely markers of the vascular status, but also of heart function, has been gaining momentum (300–303). For instance, the need to correct the AIx for different heart rates (304) points to the influence of heart function on inflection points.

Interestingly, the time at which the inflection point is found on the A-type wave hardly changes with age (305). This opposed the general view that reflections occur at one or two distinct distal reflection sites, and that with age-related increases in arterial stiffness and thus PWV, the reflected wave would return earlier. The notion of a few distinct reflection sites is probably an oversimplification; a more comprehensive analysis of wave reflection using 1-D models reconciles the findings of increasing AIx with limited changes in “reflection time” (306, 307). It is not yet clear how the arrival time of reflected waves from pressure PWs can be best determined (273, 308). Indeed, detailed model-based analysis suggests that neither the inflection point nor the shoulder point can be directly related to the return of the reflected wave (309, 310).

The ankle-brachial index (ABI) is an easily accessible and well-known indicator of the atherosclerosis component of vascular aging. It is calculated as the ratio between ankle and brachial systolic blood pressure (311). A more novel index of atherosclerosis is the ambulatory arterial stiffness index (AASI). It is assessed by a 24-h ambulatory blood pressure measurement and therefore loses the disadvantages of a snapshot measurement. AASI describes the linear relationship between systolic and diastolic blood pressure (312), however, is not so much a measure of arterial stiffness, but more a measure of ventriculo-arterial coupling determined by heart rate and vascular resistance (313, 314). The cardio-ankle vascular index (CAVI) is an advanced index reflecting the stiffness of the arterial tree. It uses PWV to deduce the parameter β (315), which was developed as a pressure-independent measure of stiffness (316). However, the pressure independence has been challenged and an improved parameter has been provided (317).

##### 3.3.2.2. Flow indices.

The flow AIx is defined similarly as pressure AIx and is calculated as the ratio between the late and early systolic velocity wave heights (Fig. 8*A*); thus, flow AIx is related to the amplitude and timing of wave reflection (318). It has been shown that carotid flow AIx is more closely associated with age, arterial stiffness parameters (such as aortic PWV, compliance, and elastic/muscular PWV ratio), and microvascular damage in brain than aortic pressure AIx (173). The aortic reverse-to-forward flow ratio has been found to be independently associated with aortic PWV and characteristic impedance, supporting the hypotheses that aortic stiffness determines the extent of flow reversal from the descending aorta to the aortic arch (175). Furthermore, aortic arteriosclerosis (assessed as reduced PPA, increased aortic PWV, and pressure augmentation) affects femoral flow wave morphology by decreasing femoral reverse-to-forward flow index and diastolic-to-systolic forward flow ratio (Fig. 8*B*) (174).

##### 3.3.2.3. PPG indices.

Several indices of vascular age can be derived from the PPG PW, as illustrated in Fig. 7*B*. These are typically calculated from the time delay or difference in amplitudes between two fiducial points. For instance, Δ*T* is the time delay between systolic and diastolic peaks (*top*), and the aging index is calculated from the amplitudes of points on the second derivative (*bottom*) as (*b − c* − *d − e*)*/a*. The wide range of PPG-based indices is reviewed in Ref. 182, and the most pertinent indices are now discussed.

The aging index has been found to correlate with carotid-femoral PWV and chronological age (176, 319), and to be associated with the presence of atherosclerosis. The aging index was designed to increase with chronological age, with correlations of *r* = 0.80 and *r* = 0.42 with age reported in the original and a subsequent publication, respectively (176, 177). Multiple studies have found that the aging index may have utility as a measure of atherosclerosis (320, 321).

The stiffness index is calculated as height*/*Δ*T*, providing a value in m/s to mimic PWV measurements. It has been found to correlate with carotid-femoral PWV (*r* = 0.65) (322), to be a genetically causal risk factor for coronary artery disease (323), and to be higher in diabetic than healthy subjects (324). The stiffness index is available in the UK Biobank Database (325), enabling extensive research into its potential utility.

The pulse rise time is the time from pulse onset to systolic peak. It has been found to be increased in subjects with peripheral arterial disease compared with healthy subjects (326), and to be increased in patients with hypertension and arteriosclerosis (327). The pulse rise time could have utility for identifying signs of peripheral arterial disease, particularly when measured at the toe.

## 4. RESEARCH DIRECTIONS

This section provides directions for future research to realize the potential of modeling and analysis of PW signals for vascular age assessment in the clinic and daily life.

### 4.1. New Generation of Cardiovascular Models

Current state-of-the-art 1-D/0-D models typically provide PWs in steady-state, supine conditions over a period of seconds. However, to unlock the full potential of reduced-order modeling for vascular age studies, future 1-D/0-D model formulations should describe the hemodynamic effects on arterial PWs of *1*) respiration, e.g., by including intrathoracic pressure as an extra variable affecting functional vessel stiffness and ventricular preload; *2*) physiological regulation by using feedback loops that dynamically adapt relevant model parameters and boundary conditions; and *3*) gravity/fluid shifts by adding source terms to the governing equations, e.g., a gravity term to the conservation of linear momentum equation. These improvements will generate beat-to-beat variations under a wide range of dynamic, transient hemodynamic conditions (e.g., horizontal rest, postural changes, mental stress, exercise, sleep) over minutes and hours. Longer-term dynamic aspects of PWs from birth to old age, including sex-specific growth patterns, adiposity gain, and CV disease progression should also be formulated and coupled to the state-of-the-art 1-D/0-D governing equations. As a result, arterial PW models could be used to simulate PW signals with growth and aging for both sexes, over a time span of years.

Arterial stiffness, which varies along the arterial network and with aging, sex, and disease, has a considerable influence on simulated pulse waveforms. It is, therefore, a key physiological parameter in 1-D models. Future models should incorporate the latest knowledge on the mechanobiological homeostasis of the arteries’ constituents (elastin, collagen, smooth muscle cells, proteoglycans) and their evolution from (pre-)birth to adulthood and throughout adulthood. They should therefore go beyond current approaches requiring detailed knowledge of arterial stiffness across the network, impeding model personalization. This new approach will also allow accounting for the impact of metabolic disorders, inflammation, or other factors (e.g., hormones, genetics), and processes on the mechanobiology of blood vessels and their material properties.

Current state-of-the-art 1-D/0-D models are deterministic. In the future, nondeterministic models should be created to account for biological variability and uncertainty in the input parameters of the models (e.g., due to measurement errors). Bayesian methods and Gaussian process regression can be used to quantify how uncertainty translates into variability in model-generated PWs (328, 329) and their predicted evolution during growth and aging. Measurement noise (81) and artifacts should also be considered to make simulated signals more realistic.

### 4.2. Unleashing the Potential of In Silico Data

The data sets of in silico PWs described in *Section 2.1.2* offer a novel and cost-effective approach for the development and preclinical testing of PW analysis algorithms across a wide range of CV conditions, in a relatively quick and inexpensive manner. Current in silico PWs have allowed us to *1*) understand the physical mechanisms underlying observations from real populations and *2*) train and test machine learning-based PW analysis algorithms; e.g., for aneurysm (81, 120) and stenosis (120) detection, arterial stiffness calculation (28, 105), and cardiac elastance assessment (330).

So far, machine learning models trained using in silico data have been tested using in silico data only. In the future, combination of in silico training of algorithms with in vivo testing in real populations could overcome the need to acquire large data sets in vivo. This will require data sets of in silico PWs created using a new generation of CV models (see *Section 4.1*) that can replicate trajectories of CV growth, remodeling, and aging in children, adolescents, and adults, for both sexes and in a wide variety of physiological and pathological conditions, including early vascular aging (4).

### 4.3. AI-Based Algorithms and Digital Twins

We envision PW analysis algorithms based on AI for vascular age assessment from PW signals acquired under varying physiological conditions in daily life, from infants to adults. AI-based algorithms could be constructed using in silico data (see *Section 4.2*) to assess arterial stiffness from basic clinical data (age, sex, body height/weight) and PW signals acquired by noninvasive wearable devices (e.g., the PPG signal). We also envision digital twins of the CV system capable of predicting an individual’s CV aging trajectory. This could lead to an early assessment of vascular age for patient stratification. Data assimilation and AI techniques will allow the new generation of CV models (see *Section 4.1*) to be used as digital twins for personalized diagnosis, prognosis, and therapy. Current state-of-the-art models require detailed anatomical and physiological data sets to estimate model parameters, yet currently rely on only a few anatomical data sets of “representative” adult males. This unworkable and biased approach should be abandoned. Instead, generation of digital twins will require data assimilation algorithms and morphing/scaling methods to generate an individual’s changing arterial network throughout life, matching body size for males/females. Existing imaging and deep phenotyping data [e.g., UK Biobank (325)] could be used for this purpose. UK Biobank also contains PW data together with epidemiological, demographic, and genomics data, which enables investigation of the genome-wide associations of PW signals, their prognostic value for incident CV disease (331), and their use in Mendelian randomization studies (332).

### 4.4. Clinical Perspectives

Ultimately, AI-based algorithms and digital twins should be tested using longitudinal studies in large populations, such as UK Biobank (331), Framingham (333), or Asklepios (334). These methods could be calibrated to the individual’s clinical and measured data at study onset. Follow-up data could then be used to assess how well the effective evolution of the subject’s CV system and the aging process of their arteries matches model predictions and, hence, facilitates the early identification of at-risk citizens. This should be compared with current practice where CV disease screening mostly requires direct contact with patients. Application of these digital solutions in children and adolescents offers the prospect to detect adverse CV trajectories with accelerated stiffening of arteries, elevated blood pressures, and concomitant cardiac problems (ventricular hypertrophy, heart failure). Preventive measures could then be targeted at high-risk individuals to protect the CV system from prolonged insults and accumulating damage that manifest as an increased CV risk in later life. A combination of population level- and high-risk-targeted prevention would represent a cost-efficient solution with a high societal impact.

## 5. CONCLUSIONS

This review has shown that modeling and analysis of arterial PWs play a key role in vascular age studies, in the clinic and in daily life. The following main conclusions arise from our review:

1)  Blood pressure, blood flow velocity, blood flow rate, arterial distension, and PPG PW signals contain a wealth of information suitable for vascular age assessment and identification of individuals at elevated CV risk.
2)  PW signals can be measured by a variety of invasive and noninvasive devices, including wearable technologies. Their characteristics and morphologies, which differ between different signal types, measurement sites, and ages need to be considered when measuring, preprocessing, and analyzing PW signals.
3)  Further research is needed to identify the most accurate PW analysis method considering the characteristics and morphology of the available input PW signal/s. Modeling can facilitate this research by providing reference data sets of in silico PWs to benchmark PW analysis methods and thereby identify reliable methods that are ready for implementation in real subjects.
4)  Physics-based, reduced-order 1-D and 0-D models can simulate PWs in large arteries, often in steady state, in supine conditions, and over one cardiac cycle, with a reasonable computational cost and with accuracies comparable with those obtained by 3-D models. However, to unlock the full potential of reduced-order modeling for vascular age studies, models should simulate PW signals under a wide range of dynamic hemodynamic conditions, accounting for uncertainty in the input parameters, biological variability, and long-term mechanobiological processes related to growth, aging, sex, and disease, from birth to old age.
5)  We envision AI-based algorithms and digital twins capable of predicting an individual’s CV aging trajectory through model-based, automated interpretation of PW signals, from frequent recordings by noninvasive, wearable technologies, throughout life. The development of these tools will require a combination of in silico and in vivo PW data, to overcome the need to acquire large data sets in vivo.

## SUPPLEMENTAL DATA

10.6084/m9.figshare.21758012.v3 Technical Supplement: https://doi.org/10.6084/m9.figshare.21758012.v3.

## GRANTS

This article is based upon work from COST Action “Network for Research in Vascular Ageing” (VascAgeNet, CA18216), supported by COST (European Cooperation in Science and Technology, www.cost.eu). This work was supported by British Heart Foundation Grants PG/15/104/31913 (to J.A. and P.H.C.), FS/20/20/34626 (to P.H.C.), and AA/18/6/34223, PG/17/90/33415, SPG 2822621, and SP/F/21/150020 (to A.D.H.); Kaunas University of Technology Grant INP2022/16 (to B.P.); European Research Executive Agency, Marie-Sklodowska Curie Actions Individual Fellowship Grant 101038096 (to S.P.); Istinye University, BAP Project Grant 2019B1 (to S.P.); “la Caixa” Foundation Grant LCF/BQ/PR22/11920008 (to A.G.); and National Institute for Health and Care Research Grant AI AWARD02499 and EU Horizon 2020 Grant H2020 848109 (to A.D.H.).

## DISCLOSURES

No conflicts of interest, financial or otherwise, are declared by the authors.

## AUTHOR CONTRIBUTIONS

J.A. and B.E.W. conceived and designed research; J.A., P.H.C., B.P., and B.E.W. prepared figures; J.A., P.H.C., V.B., B.P., B.H., A.D.H., P.S., and B.E.W., drafted manuscript; J.A., P.H.C., V.B., B.P., B.H., R.M.B., M.P.M., S.V., S.P., A.W.K., A.G., C.C.M., J.M., A.D.H., P.S., and B.E.W., edited and revised manuscript; J.A., P.H.C., V.B., B.P., B.H., R.M.B., M.P.M., S.V., S.P., A.W.K., A.G., C.C.M., J.M., A.D.H., P.S., and B.E.W. approved final version of manuscript.

## Footnotes

1 All the equation labels in the main text refer to equations found in Technical Supplement.

2 A reasonable assumption in large blood vessels (13). Non-Newtonian effects were studied by Robertson and Zakaria (14).

3 Reynolds’ numbers based on mean velocities are well below 2,000 in normal conditions (15).

4 Windkessel is German (hence the capital initial letter) for air chamber.
